# A Randomized Trial of Time-Limited Antiretroviral Therapy in Acute/Early HIV Infection

**DOI:** 10.1371/journal.pone.0143259

**Published:** 2015-11-24

**Authors:** Joseph B. Margolick, Linda Apuzzo, Joel Singer, Hubert Wong, Terry Lee, Joel E. Gallant, Phillippe El-Helou, Mona R. Loutfy, Anita Rachlis, Christopher Fraser, Kenneth Kasper, Cécile Tremblay, Harout Tossonian, Brian Conway

**Affiliations:** 1 Department of Molecular Microbiology and Immunology, Johns Hopkins Bloomberg School of Public Health, Baltimore, Maryland, United States of America; 2 CIHR Canadian HIV Trials Network, Vancouver, British Columbia, Canada; 3 Department of Medicine, Johns Hopkins School of Medicine, Baltimore, Maryland, United States of America; 4 Department of Medicine, McMaster University, Hamilton, Ontario Canada; 5 Maple Leaf Medical Clinic, Toronto, Ontario, Canada; 6 Sunnybrook Health Sciences, Toronto, Ontario, Canada; 7 Cool Aid Community Health Center, Victoria, British Columbia, Canada; 8 Department of Medical Microbiology, University of Manitoba, Winnipeg, Manitoba, Canada; 9 Centre de recherché du Centre Hospitalier de l’ Université de Montréal, Montréal, Quebec, Canada; 10 Vancouver Infectious Diseases Centre, Vancouver, British Columbia, Canada; University of New South Wales, AUSTRALIA

## Abstract

**Background:**

It has been proposed that initiation of antiretroviral treatment (ART) very soon after establishment of HIV infection may be beneficial by improving host control of HIV replication and delaying disease progression.

**Methods:**

People with documented HIV infection of less than 12 months’ duration in Baltimore MD and seven Canadian sites were randomized to either a) observation and deferred ART, or b) immediate treatment with ART for 12 months. All subjects not receiving ART were followed quarterly and permanent ART was initiated according to contemporaneous treatment guidelines. The endpoint of the trial was total ART-free time from study entry until initiation of permanent ART.

**Results:**

One hundred thirteen people were randomized, 56 to the observation arm and 57 to the immediate treatment arm. Twenty-three had acute (<2 months) infection and 90 early (2–12 months) infection. Of those randomized to the immediate treatment arm, 37 completed 12 months of ART according to protocol, 9 declined to stop ART after 12 months, and 11 were nonadherent to the protocol or lost to follow-up. Comparing those in the observation arm to either those who completed 12 months of ART or all 56 who were randomized to immediate ART, there was no significant difference between the arms in treatment-free interval after study entry, which was about 18 months in both arms.

**Conclusions:**

This study did not find a benefit from administration of a brief, time-limited (12-month) course of ART in acute or early HIV infection.

**Trial Registration:**

ClinicalTrials.gov NCT00106171

## Introduction

The rationale for treatment of HIV infection shortly after infection has frequently been considered separately from treatment of chronic HIV infection because of the possibility that the use of antiretroviral therapy (ART) in this setting could preserve anti-HIV immunity mediated by CD4- T-cells and other cells [1–5]. Other potential benefits of early treatment could include reduction of viral reservoirs [6] and of chronic inflammation and immune activation [7]. Initial studies found a clinical benefit of intervention in early HIV infection, even with monotherapy [8]. Large, nonrandomized observational studies also suggested that a short course of ART given for several months may have longer lasting benefits and delay the need for life-long ART [9]. With these considerations in mind, randomized clinical trials were initiated to address the potential benefit of a short course of ART in acute or early HIV infection; three such studies involving 12–60 weeks of ART have been reported, all showing at most a modest and transient benefit of early treatment in patients infected with HIV for up to 6 months [10–12]

We report the results of a randomized trial in which individuals with acute and early HIV infection (acquired in the previous 0–2 or 2–12 months, respectively) were randomized to be observed or to be treated with a 48-week course of ART followed by initiation (or re-initiation) of ART according to contemporaneous treatment guidelines. The goal of the study was to determine whether early ART led to a more favorable viral setpoint off treatment and/or a delay in the time to initiation of permanent ART.

## Methods

### Recruitment, randomization, and treatment

People who had been infected with HIV for less than one year were recruited from one US and seven Canadian sites (see S1 Appendix). Entry criteria were one of the following: a) detectable plasma HIV RNA with negative or indeterminate HIV antibody test results, b) positive detuned (“less sensitive”) HIV antibody ELISA test, with an optical density < 1.0 units after appropriate dilution [13], performed in the laboratory of Dr. S. Fiscus, Univ. of North Carolina) [14] which indicates a mean time of infection of approximately 6 months and a >95% likelihood of infection within the last 12 months; or c) positive HIV antibody test result with a documented negative result within the previous 12 months. Participants were considered to have acute infection (duration of 2 months or less) if they met criterion a) or had a documented negative HIV antibody test result within 2 months of the positive test; otherwise they were considered to have early infection (duration 2–12 months). Eligibility criteria included age 18 years and over, willingness to give written informed consent, ability to swallow tablets/capsules, willingness to use barrier methods of contraception, no prior use of ART (other than post-exposure prophylaxis), CD4+ cell count >350 cells/μL (if infected >3 months), and plasma HIV RNA >5,000 copies/mL (if infected > 6 months). Required laboratory values (within 28 days of randomization) included: absolute neutrophil count ≥750 cells/μL; hemoglobin ≥70 g/L; platelet count ≥50,000/μL; creatinine ≤3 x upper limit of normal (ULN); AST (SGOT), ALT (SGPT), and alkaline phosphatase ≤5 × ULN; total bilirubin ≤2.5 x ULN; and a negative serum pregnancy test in women of childbearing potential. Exclusion criteria were inability to design an ART regimen based on the results of pre-treatment genotypic resistance testing; pregnancy or breast feeding; receipt of systemic cancer chemotherapy, systemic investigational agents, immunomodulators (growth factors, systemic corticosteroids, HIV vaccines, immune globulin, interleukins, interferons) within 28 days prior to study entry; and acute illness requiring systemic treatment and/or hospitalization (the subject was eligible ≥7 days after resolution of the illness). Active recreational drug or alcohol use was exclusionary only if it would interfere with adherence to study requirements in the opinion of the investigator. The trial was registered at clinicaltrials.gov as Clinical Trials registration #: NCT00106171 and all ongoing or related trials were registered. The full protocol of the study is available with the online version of this article.

Subjects were enrolled from May, 2005 to September, 2009, and upon enrollment were randomized 1:1 either to receive ART for 12 months beginning immediately or to be observed until ART was clinically indicated based on contemporaneous treatment guidelines. Random allocation lists were prepared by a statistician unassociated with the study using computerized software. Allocation was stratified by center and by duration of infection (acute or early), with random permuted blocks of size two and four. Randomization was performed by dialing into a secure computer-based system located at the CIHR Canadian HIV Trials Network data coordinating center in Vancouver, Canada which recorded all events for accuracy and verification. Specific ART regimens were selected by the subject’s clinician, guided by current best practices and the results of resistance testing.

After randomization, all study subjects were evaluated on a monthly basis, with measurement of plasma HIV RNA each month and CD4 cell count assessment quarterly for the first 12 months; thereafter subjects were evaluated quarterly (or more frequently as clinically indicated), including measurements of both CD4 cell count and plasma HIV RNA. Participants receiving ART completed an adherence questionnaire, and strategies aimed at enhancing adherence were discussed at each study visit. Regimens were changed as needed to support optimal adherence and address medication-related side effects. When contemporary guidelines for initiation of ART were met (CD4 cell count <350/mm^3^ at all times, and <500/mm^3^ beginning in 2010 for U.S. subjects), permanent ART was initiated for subjects in either arm who were not receiving ART. Follow-up was completed in May, 2012; at that time the trial was stopped due to completion of available data on outcomes.

For both arms, all study visits included assessment of new or ongoing diagnoses, adverse events, concomitant medications, a targeted physical examination, assessment of risk behaviors, and routine laboratory assessments.

### Laboratory studies

T cell subsets were measured by flow cytometry and HIV viral loads by RT-PCR at each site’s local laboratories using FDA-approved assays with a limit of quantitation of 50 copies/mL of plasma or less. Viral load testing at entry and 24 and 36 months after entry was required to be performed by laboratories certified by NIH-sponsored quality control programs; the test used for these time points was Roche Amplicor v. 1.5 (Roche Diagnostics, Nutley, NJ) in the U.S. and Roche Taqman (IRoche), Roche Amplicor (Roche), or Chiron bDNA 3.0 (Chiron, Emeryville, CA) in Canada.

### Outcome definitions

Initially, the planned primary outcome of the trial was the comparison of plasma HIV RNA at 24 months off treatment in the two study groups (i.e., month 24 for the deferred treatment arm and month 36 for the immediate treatment arm). However, it became evident during the study that disease progression off ART was more rapid than expected and that many, if not most, would be initiating ART before 24 months off treatment. While the intention was to compare viral load reflected by natural, non-interventional processes, the viral load at 24 months was, in fact, influenced by intervention with ART in the majority of individuals, and was not in concert with the aims of the study. Therefore, in consultation with the CIHR Canadian HIV Trials Network Data and Safety Monitoring Board and the Division of AIDS of NIH, the primary outcome was changed to total treatment-free time before initiation of permanent ART, i.e., time from study entry to initiation of ART in the deferred treatment arm, and time from cessation of ART to its permanent resumption in the immediate treatment arm.

### Sample Size

The original study was powered on the basis of an expected difference of .25 log_10_ copies/mL with a standard deviation of .5 log_10_ copies/mL at 12 and 24 months after the end of the 12-month randomization period during which one arm was treated and the other was not. To detect this difference with a two-tailed alpha of .05 and a power of .8, we required 64 patients per arm. However, we decided to try to recruit 180 patients in order to increase the power for the main comparison to 90%, and to maintain a power of .8 if the observed standard deviation turned out to be as high as .6 log_10_ copies/mL. When it became apparent that a much higher than expected proportion of study participants were initiating antiretroviral therapy within the follow-up period, thus making the viral load outcome irrelevant, the study investigators, with approval of the Data Safety Monitoring Board of the CIHR Canadian HIV Trials Network, revised the primary endpoint to ART-free years. The sample size was recalculated to be 60 per treatment arm to be able to detect, with a power of .8, a difference in median HAART-free years from 1.8 in the control group to 3.8 years in the immediate treatment group, assuming a median follow-up time of 2.5 years.

### Statistical analysis

For crude analysis, Kaplan-Meier curves were used to describe the distributions of treatment-free time before initiation of permanent ART in the two arms. Time 0 was set to study entry in the deferred treatment arm and to time of cessation of immediate ART in the other arm. The Cox proportional hazards model was employed to assess the difference between the two arms both on an intent-to-treat (ITT) basis and on a per-protocol (PP) basis. These analyses adjusted for sex, stage of infection at study entry (i.e., acute vs. early), age, injection drug use, and whether baseline viral load was >10,000 copies/mL. For the ITT analysis, multiple imputation by chained equations was used to impute the time to initiation of permanent therapy (or censoring) for subjects in the immediate arm who stopped ART during the first 48 weeks, were lost to follow-up before 48 weeks, or chose not to discontinue ART at 48 weeks. Baseline demographics and virologic and immunologic parameters were used as auxiliary variables in the imputation process. For the PP analysis, subjects in the immediate treatment arm who stopped ART prior to 48 weeks, were lost to follow-up before 48 weeks, or chose not to discontinue ART at 48 weeks were excluded because they did not complete and stop the immediate treatment mandated by the study protocol. The proportions of participants in each arm who achieved 24 months of ART-free time, and their plasma viral loads at that time, were compared between the two arms in univariate analysis using a Fisher’s exact test and two-sample t-test, respectively, and in multivariable analysis using linear regression with adjustment for the same baseline variables included in the Cox analyses.

### Ethics Statement

This study was approved by institutional review boards at all participating sites, as follows: the Johns Hopkins Bloomberg School of Public Health Institutional Review Board, IRB No: H.26.04.03.17.A1; the University of British Columbia Clinical Research Ethics Board, No: H05-70428; Sunnybrook Research Ethics Board, No: 389–2006; Quebec Research Ethics Board approved the study on April 3, 2007; University of Manitoba Biomedical Research Ethics Board, No. B2006:115; Comite D’Equique de la Recherche Du Chum No: SL06.093. Written informed consent was provided by all study participants.

## Results

### Study participants

One hundred thirteen subjects were randomized, 57 to the immediate and 56 to the deferred treatment arm (**Table 1**). Participants were predominantly male and Caucasian; approximately one-fifth had acute HIV infection. Route of infection was predominantly through male-male sexual contact; approximately one-fifth of subjects in each arm acquired HIV through injection drug use and heterosexual contact. The arms were similar in these respects, and also in age and baseline HIV plasma viral load. The subjects in the immediate treatment arm were slightly older and more likely to be African-American, and had slightly lower CD4 and CD8 T cell counts. Baseline plasma viral load was higher in the acute (60.9% >10,000 copies/ml) than in the early patients (16.7%).

**Table 1:** Demographics of Study Participants.

				Immediate Arm	
Variable	All (n = 113)	Deferred Arm (n = 56)	Immediate Arm (n = 57)	Completed and stopped ART (n = 37)	Didn't stop or didn’t complete ART (n = 20)
Sex					
Male	99 (87.6)	47 (83.9)	52 (91.2)	34 (91.9)	18 (90.0)
Female	14 (12.4)	9 (16.1)	5 (8.8)	3 (8.1)	2 (10.0)
Stratum					
Acute	23 (20.4)	13 (23.2)	10 (17.5)	6 (16.2)	4 (20.0)
Early	90 (79.6)	43 (76.8)	47 (82.5)	31 (83.8)	16 (80.0)
Race					
Black or African American	28 (24.8)	10 (17.9)	18 (31.6)	9 (24.3)	9 (45.0)
White	75 (66.4)	40 (71.4)	35 (61.4)	26 (70.3)	9 (45.0)
Other	10 (8.8)	6 (10.7)	4 (7.0)	2 (5.4)	2 (10.0)
Ethnicity					
Hispanic or Latino	2 (1.8)	1 (1.8)	1 (1.8)	1 (2.7)	0 (0.0)
Not Hispanic or Latino	109 (96.5)	55 (98.2)	54 (94.7)	36 (97.3)	18 (90.0)
Not available to clinic	2 (1.8)	0 (0.0)	2 (3.5)	0 (0.0)	2 (10.0)
Route of transmission					
Injection drug use (current or previous)*	24 (21.6)	13 (23.2)	11 (20.0)	9 (25.0)	2 (10.5)
MSM	80 (70.8)	36 (64.3)	44 (77.2)	30 (81.1)	14 (70.0)
Heterosexual contact	29 (25.7)	16 (28.6)	13 (22.8)	8 (21.6)	5 (25.0)
Age (yr)					
Median (IQR)	33.2 (26.7, 43.1)	32.8 (27.2, 44.4)	34.7 (26.1, 42.3)	36.8 (31.7, 46.5)	27.7 (24.5, 35.2)
Range	(18.9, 66.7)	(18.9, 66.7)	(19.0, 58.4)	(19.3, 58.4)	(19.0, 56.1)
Baseline CD4 cell count (/ul)					
Median (IQR)	530 (430, 650)	556 (433, 655)	506 (420, 650)	500 (382, 650)	533 (430, 652.5)
Range	(168, 1483)	(168, 1483)	(220, 900)	(237, 900)	(220, 770)
Baseline CD8 cell count (/ul)					
Median (IQR)	915 (740, 1268)	953 (732, 1420)	910 (740, 1160)	940 (772, 1240)	880 (705, 1045)
Range	(166, 6369)	(491, 3004)	(166, 6369)	(166, 6369)	(530, 1508)
Baseline viral load (log_10_ copies/ml)					
Median (IQR)	4.6 (4.1, 5.1)	4.6 (4.0, 5.1)	4.7 (4.2, 5.0)	4.6 (4.2, 5.0)	4.7 (4.2, 4.9)
Range	(2.4, 6.6)	(2.4, 5.9)	(3.1, 6.6)	(3.1, 6.6)	(3.5, 5.9)
Site					
Hotel Dieu	1 (0.9)	0 (0.0)	1 (1.8)	1 (2.7)	0 (0.0)
Sunnybrook	6 (5.3)	3 (5.4)	3 (5.3)	2 (5.4)	1 (5.0)
Hamilton	4 (3.5)	2 (3.6)	2 (3.5)	1 (2.7)	1 (5.0)
Maple Leaf	6 (5.3)	3 (5.4)	3 (5.3)	0 (0.0)	3 (15.0)
Vancouver	28 (24.8)	15 (26.8)	13 (22.8)	9 (24.3)	4 (20.0)
Victoria	6 (5.3)	3 (5.4)	3 (5.3)	3 (8.1)	0 (0.0)
Winnipeg	6 (5.3)	4 (7.1)	2 (3.5)	1 (2.7)	1 (5.0)
Baltimore	56 (49.6)	26 (46.4)	30 (52.6)	20 (54.1)	10 (50.0)

* Data missing for 2 patients

### Conduct of study protocol

**Fig 1:**
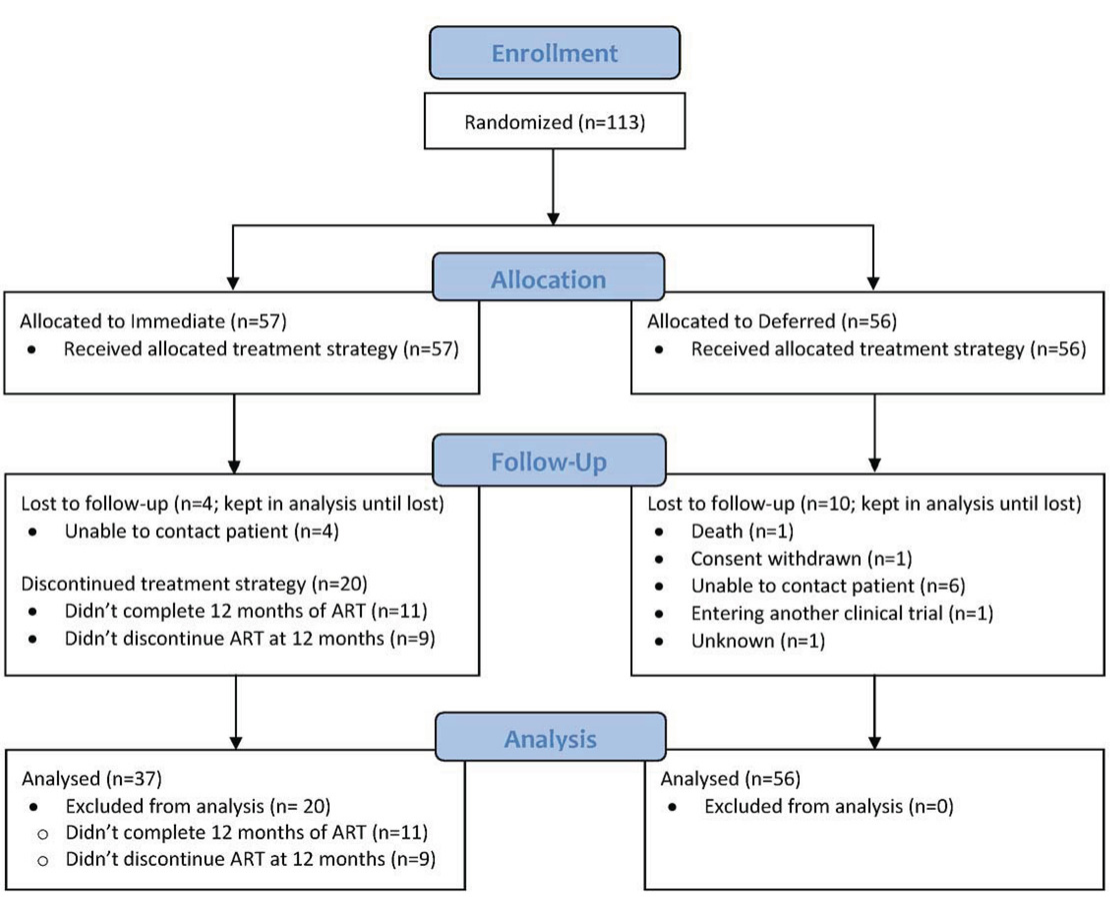
Disposition of randomized study participants.

Disposition of randomized participants is shown in **Fig 1.** Loss to follow-up was similar in both arms. Of the 57 participants in the immediate treatment arm, 46 (81%) completed 12 months of ART and 11 (19%) were either non-adherent to therapy or lost to follow-up. No information on the subsequent treatment or outcomes of these 11 participants are available. Of those who completed 12 months of ART, 9 chose not to discontinue ART at 48 weeks, as mandated by the study protocol, leaving 37 who adhered to the protocol and were included in the per-protocol analysis. Virologic and immunologic parameters were similar between those who did or did not stop ART at 48 weeks (**S1 Table**), but the latter were somewhat younger than the former (median 29.5 yrs (IQR 26.1–34.7) vs. 36.8 (31.7–46.5), respectively). Decisions not to stop ART were idiosyncratic and unrelated to effects of medications or to the change in treatment guidelines (data not shown). Adverse events were uncommon in both arms and there were no medication-related serious adverse events.

In the immediate arm, all of the 37 subjects who stopped ART after 12 months achieved full viral suppression while on initial therapy, 36/37 at three or more time points. The median duration of suppression was 7.9 months (IQR = 5.6–9.0 months). Duration of suppression was significantly and inversely related to baseline plasma viral load, but not to sex, duration of infection (acute vs. early), injection drug use, route of infection, age, or baseline CD4 cell count (data not shown). All but 6 subjects had undetectable plasma viral load at the end of therapy, and 5 of these were <1000 copies/ml. After stopping ART, 36 of the 37 subjects had confirmed viral rebound within 3 months; the remaining subject was not tested within this interval.

### Initiation or reinitiation of therapy

**Fig 2:**
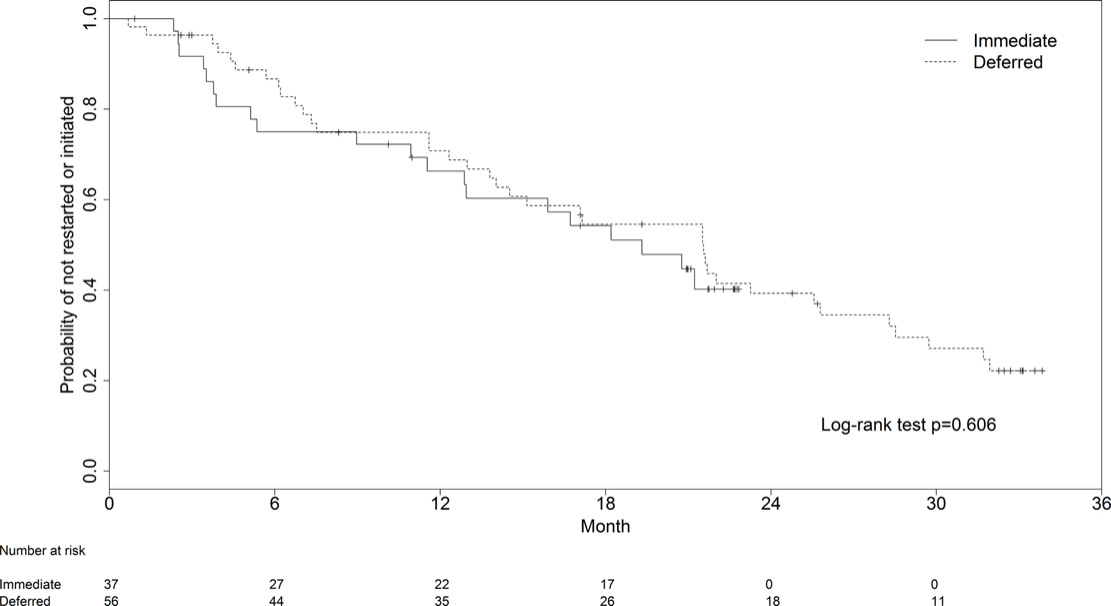
Per-protocol survival analysis of time from randomization (Deferred Treatment arm) or cessation of immediate ART (Immediate Treatment arm) until permanent initiation of antiretroviral therapy (ART). Results are stratified by study arm (Immediate ART vs. Deferred ART). The Immediate ART arm includes the 37 subjects who completed the study protocol, i.e., 12 months of ART followed by cessation of ART and continuation of clinical monitoring. Numbers of observations at 6-monthly time points are shown. (For the results of the same analysis including the 9 subjects in the Immediate arm who completed 12 months of ART but did not stop ART at that time, see S1 Fig).

In both the immediate and deferred arms, the median treatment-free time before initiation of permanent ART was approximately 18 months **(Fig 2)**, and the arms did not differ significantly in this regard. In the Cox analyses with adjustment for baseline variables **(Table 2)**, treatment-free time remained not significantly different between the two arms in both the ITT analysis [HR = 0.95; 95% CI: 0.51, 1.78] and the PP analysis [HR = 0.89; 95% CI: 0.48, 1.63]. However, in the immediate arm patients with higher baseline viral load had significantly shorter time to reinitiation of therapy after adjustment for other baseline variables (p<0.01). The average rebound viral load on cessation of ART was not significantly correlated with time to reinitiation of ART (r = 0.29 using the log_10_ scale; p = 0.22). The median CD4 cell counts at initiation of permanent ART did not differ significantly between the two study arms: 365 (290, 597) cells/ul for the Immediate arm, and 334 (280, 407) for the Deferred arm (p = 0.198). Subgroup analysis by stage of infection did not show any significant difference between the two arms in either the ITT analysis [HR = 1.14; 95% CI: 0.25, 5.18 in the acute group; HR = 0.85; 95% CI: 0.43, 1.70 in the early group] or the PP analysis [HR = 0.65; 95% CI: 0.16, 2.64 in the acute group; HR = 0.88; 95% CI: 0.44, 1.78 in the early group].

**Table 2:** Adjusted effect of immediate cART on treatment-free time before initiation of permanent ART.

	Per-protocol analysis		ITT analysis	
	Hazard ratio (95% CI)	p	Hazard ratio (95% CI)	p
Treatment group				
Immediate	referent	-	referent	-
Delay	0.89 (0.48,1.63)	0.698	0.95 (0.51, 1.78)	0.872
**Adjustment variables**				
Sex				
Male	referent	-	referent	-
Female	1.15 (0.45, 2.93)	0.768	1.34 (0.53, 3.41)	0.534
Stratum				
Acute	referent	-	referent	-
Early	0.89 (0.44, 1.79)	0.744	1.14 (0.55, 2.37)	0.713
Injection drug use				
No	referent	-	referent	-
Yes	0.48 (0.23, 1.00)	0.049	0.48 (0.22, 1.03)	0.061
Age				
<25	referent	-	referent	-
25–44	1.58 (0.74, 3.34)	0.236	1.26 (0.60, 2.65)	0.545
45+	1.03 (0.43, 2.47)	0.954	0.94 (0.40, 2.24)	0.892
Baseline VL				
≤10,000	referent	-	referent	-
10,001–100,000	5.21 (1.82, 14.94)	0.002	4.77 (1.70, 13.41)	0.004
>100,000	6.90 (2.21, 21.56)	<0.001	7.01 (2.28, 21.53)	<0.001

We also performed a sensitivity analysis in which the 9 patients in the immediate group who chose to continue therapy at 12 months were considered to be immediate failures. This analysis tends to exaggerate the effect of immediate ART in a negative way, because at least some time would have passed before these patients met the criteria for permanent resumption of treatment. In this analysis, the deferred arm tended to have a longer median time to reinitiation of therapy (21.6 vs. 12.9 months; **S1 Fig**), but the results were again not statistically significant (p = 0.07). In addition, the baseline covariates that were significantly correlated with outcome did not change. Subgroup analysis by stage of infection again indicated no significant difference between the two treatment arms.

### Plasma viral load after 24 months without treatment

The planned 24-month time point to analyze viral load without ART occurred 24 months following study entry for the deferred treatment arm, and 36 months following study entry in the immediate arm. At this time point, 23 subjects in the deferred arm and 13 in the immediate arm had not yet initiated permanent ART. Among these participants, plasma viral load at this time point was lower in the immediate arm (mean 3.87 log_10_ copies/mL [SD 0.40]) than in the deferred arm (mean 4.18 log_10_ copies/mL [SD 0.74]). This difference was not significant in both univariate analysis (p = 0.17) and multivariable analysis adjusted for baseline variables mentioned above (0.11 log_10_ copies/ml higher [95% CI: -0.39, 0.62] in the deferred arm, p = 0.65). There were insufficient people with acute infection in this group (n = 1) to be able to evaluate the effect of stage of infection (i.e., acute vs. early) on this outcome.

Being off ART and having a plasma viral load <10,000 copies/ml at month 24 was twice as common among those randomized to the immediate treatment arm and stopping ART at 12 months (9/37 [24%]) as among those randomized to the deferred arm (7/56 [12%]) but this difference was not statistically significant (p = 0.16).

## Discussion

This study evaluated the benefit of a time-limited course of ART in patients recently infected with HIV. The central hypothesis was that this would lead to improved virologic and immunologic set points that would allow a meaningful delay in the permanent initiation of ART. Despite enrolling over 55 patients per arm, no benefit was shown. In fact, most patients in both study arms had started permanent ART within 2 years of study entry (or of ART cessation in those randomized to immediate treatment). Among the 36 patients who remained off ART at the end of the pre-planned period of observation, the 13 who had received a 12-month course of ART did not exhibit a more favorable immunologic or virologic profile. The results were the same whether we included or excluded the 9 people in the immediate treatment arm who chose, for reasons not related to therapy, not to stop ART at the end of the 12-month immediate treatment arm. The change in clinical guidelines for the use of ART during the period of study conduct also was very unlikely to have affected the findings of the trial, because a) most primary outcomes had occurred by this time, and b) both arms of the study had small and similar numbers of participants who initiated ART under the new guidelines.

The results of this study are generally in agreement with the three other large randomized trials that have addressed the question of whether a time-limited course of ART is beneficial in acute or early HIV infection. In the SETPOINT study, conducted primarily in the United States [10], 130 people infected for up to 6 months were randomized to immediate treatment for 36 weeks or to observation. As most people in the deferred treatment arm met criteria for initiation of ART within 18 months, the study was terminated prematurely and found only a modest benefit of immediate treatment. In the Dutch PRIMO-SHM study [11], participants were randomized to either observation or to immediate treatment for either24 or 60 weeks. 115 people were randomized, most infected for 3 months or less. Initiation of ART in the deferred arm occurred significantly sooner (median 0.7 years) than in the immediate arms (3.0 and 1.8 years, respectively), but the time from infection to permanent initiation of ART was shorter than was expected, and the benefit of immediate ART was modest at best. In the European SPARTAC study [12], 366 people infected for up to one year (most <6 months) were randomized to immediate treatment (for 12 or 48 weeks) or deferred treatment; those who received 48 weeks of immediate ART had about a 17-week benefit in total ART-free time compared to the deferred treatment group. Thus, none of these studies found a clinically important benefit of a time-limited course of ART during acute or early HIV infection, in populations that consisted primarily of people who had been infected with HIV for less than six months. The main finding of all of the studies was that after HIV infection, the natural history of HIV disease is such that most patients would have required ART within two years based on CD4 cell count criteria in use at the time, whether or not they received an immediate course of ART at the time of initial diagnosis. Our study confirms and extends these findings.

As in the present trial, previous trials were designed based on the hypothesis that limiting the immunological damage that begins immediately after HIV infection might allow the host to develop more effective anti-HIV responses that could limit viral replication. More recently, treatment interruption has fallen into disfavor for several reasons. First, recent evidence indicates that ongoing HIV replication and the associated inflammation are harmful in themselves, not only to the immune system but also because they may increase the risk of long-term non-AIDS complications of chronic inflammation and immune activation, such as cardiovascular disease [15]. Second, treatment regimens have become much simpler, and less toxic, reducing the downside of early permanent ART to the point that US guidelines now recommend initiation of ART regardless of CD4 cell count. Finally, effective ART dramatically reduces the risk of HIV transmission [16]. Since most patients will need lifelong ART beginning at the time of diagnosis, there is now little rationale for approaches that delay the initiation of permanent ART by a short time.

There is still the possibility that a small subset of people with acute or early HIV infection may benefit from an early time-limited course of ART. One such subset is patients identified within a few weeks of infection [6,17,18] or immediately after birth [19]. However, identifying such patients is exceedingly challenging, and designing programs to treat such patients is unlikely to be cost-effective. The present study suggests a possible benefit of early therapy in terms of lowering virologic set point, but this effect was achieved in only a small minority of patients, was not statistically significant, and is of uncertain clinical importance.

The present study and the other randomized studies that have addressed early, time-limited ART in acute or early HIV infection have all depended heavily on patients who presented for medical care. Patients who are symptomatic from acute HIV infection are likely to experience more rapid progression of disease than those who are asymptomatic or have mild symptoms following initial exposure to the virus [20]. Thus, it remains possible that individuals who did not participate in any of these studies because they did not seek medical care might benefit more from a course of early ART in a way that studies conducted to date have not demonstrated. However, this is speculative, and identifying and treating such patients is not feasible.

Although the question of how and whether to treat acute and early HIV infection has become less important as a clinical problem, important issues remain. There is a potentially important public health benefit to identification and treatment of HIV-infected individuals as soon as possible after infection. Such patients often have extremely high levels of viremia, which increases the risk of transmission of HIV; treating them at the earliest opportunity may greatly reduce this risk [21]. Guidelines recommend treatment of early HIV infection, and more and more practitioners are instituting it as soon as the patient is ready for it. This approach is likely to gain more favor given the simplicity and low toxicity of current ART regimens.

In summary, our study does not support the systematic use of a short course of ART in patients diagnosed with acute or early HIV infection. We believe that permanent initiation of ART should be considered from the time of initial diagnosis, both for individual benefit and to decrease the risk of transmission to others.

## Supporting Information

S1 CONSORT Checklist Consort Checklist. (DOCX)

S1 Appendix Sites in This Study and Their Primary Investigators. (DOCX)

S1 Fig Per-protocol survival analysis of time from randomization (Deferred Treatment arm) or cessation of immediate ART (Immediate Treatment arm) until permanent initiation of antiretroviral therapy (ART). The analysis and figure are the same as in Fig 2, except that the 9 subjects who completed 12 months of ART in the Immediate arm but did not stop ART at that time were considered as having initiated permanent ART at time 0.
(TIFF)

S1 Protocol Trial protocol September 2004. This document consists of the protocol that was used for this trial.
(DOCX)

S1 Table Association between successful completion of TL-cART and demographic variables. (DOCX)
