# Supplementary material for: A Randomized Trial of Time-Limited Antiretroviral Therapy in Acute/Early HIV Infection
Source: PLoS One. 2015 Nov 24;10(11):e0143259. doi: 10.1371/journal.pone.0143259 (PMC4658016; doi:10.1371/journal.pone.0143259)
Supplement: S1 Appendix — (DOCX) [file pone.0143259.s002.docx]

S1 Appendix. Sites in This Study and Their Primary Investigators

Sites included Johns Hopkins Bloomberg School of Public Health, Baltimore, MD, USA (J. Margolick, L. Apuzzo, J. Gallant); Downtown Infectious Diseases Clinic, Vancouver BC (B. Conway); Cool Aid Community Health Center, Victoria, BC (C. Fraser); Sunnybrook and Women’s College Health Sciences Center, Toronto, ON (Anita Rachlis); Hotel-Dieu du CHUM, Montreal, QC (C. Tremblay); Maple Leaf Medical Clinic, Toronto, ON (M. Loutfy); Hamilton Health Sciences Centre, Hamilton, ON (P. El-Helou); and the University of Manitoba Health Sciences Centre, Winnipeg, MB (K. Kasper).
