# Supplementary figures and images for: A Randomized Trial of Time-Limited Antiretroviral Therapy in Acute/Early HIV Infection
Source: PLoS One. 2015 Nov 24;10(11):e0143259. doi: 10.1371/journal.pone.0143259 (PMC4658016; doi:10.1371/journal.pone.0143259)

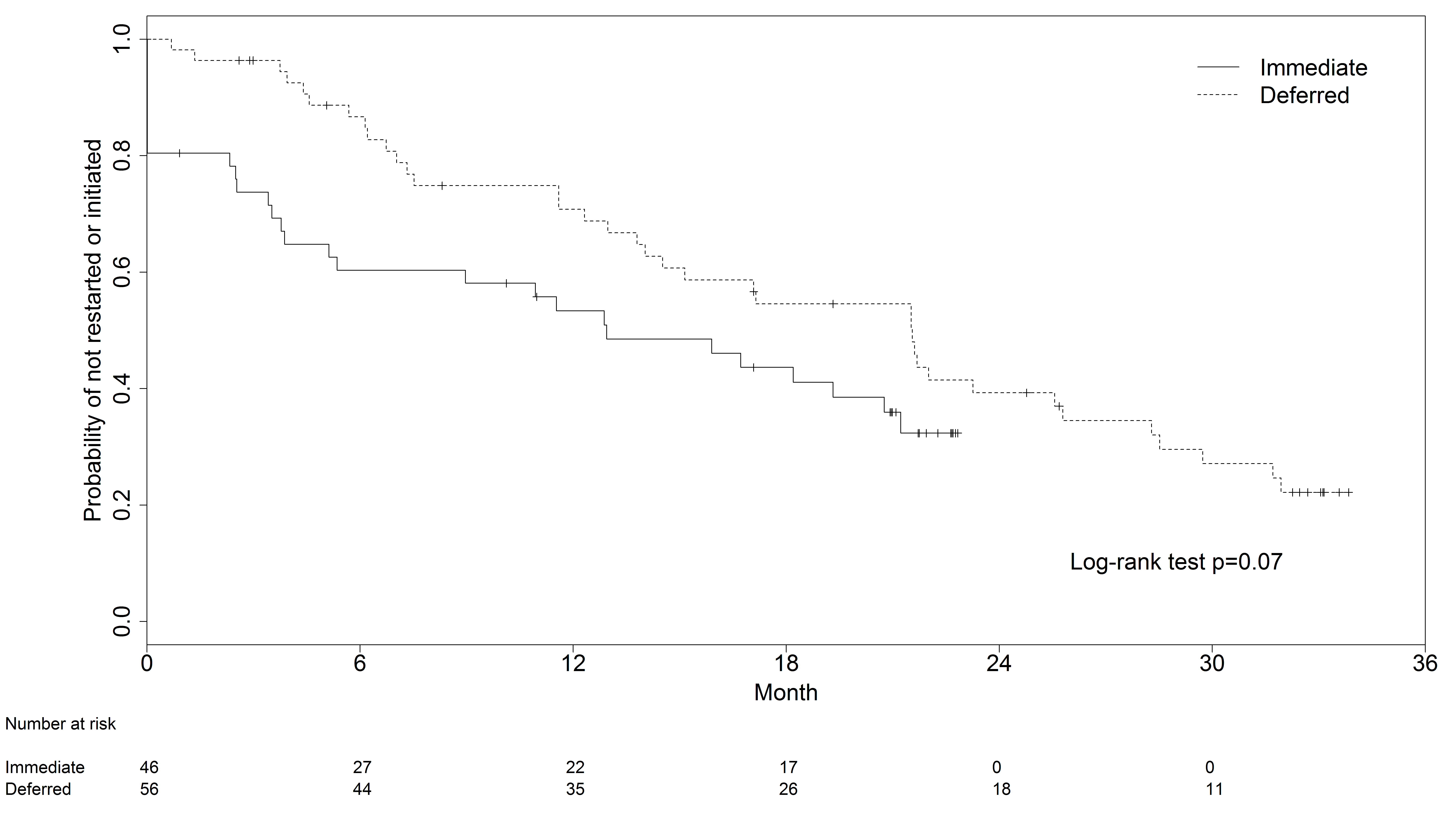

Supplement: S1 Fig — The analysis and figure are the same as in Fig 2, except that the 9 subjects who completed 12 months of ART in the Immediate arm but did not stop ART at that time were considered as having initiated permanent ART at time 0. (TIFF) [file pone.0143259.s003.tiff]
