# Supplementary material for: A Randomized Trial of Time-Limited Antiretroviral Therapy in Acute/Early HIV Infection
Source: PLoS One. 2015 Nov 24;10(11):e0143259. doi: 10.1371/journal.pone.0143259 (PMC4658016; doi:10.1371/journal.pone.0143259)
Supplement: S1 Protocol — This document consists of the protocol that was used for this trial. (DOCX) [file pone.0143259.s004.docx]

**Phase IV Randomized, Open Label Controlled Trial of Time-Limited Highly Active Antiretroviral Therapy (TL-HAART) in Patients with Acute or Early HIV Infection**

**Sponsored by:**

**The National Institute of Allergy and Infectious Diseases**

**This is a non-IND Protocol**

**Protocol Chair: Joseph B. Margolick MD, PhD**

**Johns Hopkins University**

**Bloomberg School of Public Health**

**Department of Molecular Microbiology and Immunology**

**615 North Wolfe St E5153**

**Baltimore MD 21205**

**Telephone: 410-955-1436**

**Fax: 410-614-8263**

**jmargoli@jhsph.edu**

**Protocol Co-Chair: Brian Conway MD, FRCPC**

**University of British Columbia**

**Department of Pharmacology & Therapeutics**

**201-1200 Burrard St**

**Vancouver BC, CANADA V6Z2C7**

**Telephone: 604-642-6429**

**Fax: 604-642-6419**

**bconway@interchange.ubc.ca**

DAIDS Clinical Representative: Marjorie Dehlinger RN, DNSc

**Version 1.3**

***August 5, 2004***

TABLE OF CONTENTS

Page

SITES PARTICIPATING IN THE MAIN STUDY 4

PROTOCOL TEAM ROSTER 5

STUDY MANAGEMENT 6

SCHEMA 8

1.0 HYPOTHESIS AND STUDY OBJECTIVES 9

1.1 Hypothesis 9

1.2 Primary Objectives 9

1.3 Secondary Objectives 9

2.0 INTRODUCTION 9

2.1 Background 9

2.2 Rationale 11

3.0 STUDY DESIGN 12

4.0 SELECTION AND ENROLLMENT OF SUBJECTS 14

4.1 Inclusion Criteria 14

4.2 Exclusion Criteria 15

4.3 Study Enrollment Procedures 16

5.0 STUDY TREATMENT (OR INTERVENTION) 16

5.1 Regimens, Administration, and Duration 16

5.2 Management of inadequate viral suppression 17

5.3 Product Formulation and Preparation 19

5.4 Product Supply, Distribution, and Pharmacy 19

5.5 Concomitant Medications 19

5.6 Adherence Assessment 19

6.0 CLINICAL AND LABORATORY EVALUATIONS 20

6.1 Timing of Evaluations 22

6.2 Special Instructions and Definitions of Evaluations 21

6.3 Off-Drug Requirements 25

7.0 TOXICITY MANAGEMENT 25

8.0 CRITERIA FOR TREATMENT DISCONTINUATION 25

9.0 STATISTICAL CONSIDERATIONS 26

9.1 General Design Issues 26

9.2 Endpoints 26

9.3 Randomization and Stratification 27

9.4 Sample Size and Accrual 27

9.5 Monitoring 30

9.6 Analyses 30

10.0 PHARMACOLOGY PLAN 31

10.1 Pharmacology Objectives 31

10.2 Pharmacology Study Design 31

10.3 Primary and Secondary Data, Modeling, and Data Analysis 31

10.4 Anticipated Outcomes 31

11.0 DATA COLLECTION AND MONITORING AND ADVERSE EXPERIENCE REPORTING 31

11.1 Records to Be Kept 31

11.2 Role of Data Management 32

11.3 Clinical Site Monitoring and Record Availability 32

11.4 Serious Adverse Experience (SAE) Reporting 32

12.0 HUMAN SUBJECTS 33

12.1 Institutional Review Board (IRB) Review and Informed Consent 33

12.2 Subject Confidentiality 33

12.3 Study Discontinuation 34

13.0 PUBLICATION OF RESEARCH FINDINGS 34

14.0 BIOHAZARD CONTAINMENT 34

15.0 REFERENCES 35

APPENDIX I: SAMPLE INFORMED CONSENT

APPENDIX II: SCHEDULE OF EVALUATIONS

APPENDIX III: DIVISION OF AIDS, TABLE FOR GRADING SEVERITY OF ADULT ADVERSE EXPERIENCES

APPENDIX IV: AIEDRP ADHERENCE QUESTIONAIRE

APPENDIX V: AIEDRP STAGING ALGORITHM

# SITES PARTICIPATING IN THE MAIN STUDY

**United States**

Dr. Joseph B. Margolick

Dr. Joel E. Gallant

Dr. Patricia Barditch-Crovo

##### Johns Hopkins University,

##### Bloomberg School of Public Health

Department of Molecular Microbiology and Immunology

##### 615 North Wolfe St E5153

Baltimore MD 21205

**Canada**

Dr. Brian Conway

Dr. John Farley

Dr. Robert Reynolds

Downtown Infectious Diseases Clinic

201-1200 Burrard St

Vancouver BC CANADA V6Z 2C7

Dr. Stanley DeVlaming

Pender Community Health Center

59 West Pender St

Vancouver BC V6B 1R3

Dr. Chris Fraser

Cool Aid Community Health Center

102-749 Pandora Ave

Victoria BC V8W

1N9

Dr. Anita Rachlis

Sunnybrook and Women’s College Health Sciences Center

2075 Bayview Avenue, Room A226

Toronto ON M4N 3M5

Dr. Pierre Côté

Clinique Médicale du Quartier Latin

905 Boul. Rene-Levesque E.

Montreal QC H21 5B1

#

# PROTOCOL TEAM ROSTER

Protocol Chair

**Joseph B. Margolick MD, PhD**

**Johns Hopkins University**

**Bloomberg School of Public Health**

**Department of Molecular Microbiology and Immunology**

**615 North Wolfe St E5153**

**Baltimore MD 21205**

**Telephone: 410-955-1436**

**Fax: 410-614-8263**

**jmargoli@jhsph.edu**

Co-Chair

**Brian Conway MD, FRCPC**

**University of British Columbia**

**Department of Pharmacology & Therapeutics**

**201-1200 Burrard St**

**Vancouver BC, CANADA V6Z2C7**

**Telephone: 604-642-6429**

**Fax: 604-642-6419**

**bconway@interchange.ubc.ca**

DAIDS Clinical Representative

**Marjorie Dehlinger, RN, DNSc**

Statistician(s)

**Dr. Joel Singer**

**Canadian HIV Trials Network**

Data Manager

**Dr. Hubert Wong**

**Canadian HIV Trials Network**

Investigators

**Dr. Pierre Côté**

**Dr. Stanley DeVlaming**

**Dr. John Farley**

**Dr. Chris Fraser**

**Dr. Anita Rachlis**

**Dr. Robert Reynolds**

**Dr. Joel Gallant**

**Dr. Patricia Barditch-Crovo**

Project Directors

**Linda Apuzzo**

[**lapuzzo@jhsph.edu**](mailto:lapuzzo@jhsph.edu)

**Jennie Prasad**

**jenniepd@interchange.ubc.ca**

# STUDY MANAGEMENT

All questions concerning this protocol should be sent via e-mail to Linda Apuzzo and Jennie Prasad at [protocol@.tl-haartstudy.org](mailto:protocol@.tl-haartstudy.org). A response should generally be received within 24 hours (Monday-Friday).

Sites registering to this study should contact the Computer Support Group at the Data Management Center via e-mail ([actg.user.support@fstrf.org](mailto:actg.user.support@fstrf.org)) to have the relevant personnel at the site added to the protocol e-mail group as soon as possible. Inclusion in the protocol e-mail group will ensure that sites receive important information about the study during its implementation and conduct.

FOR QUESTIONS CONCERNING CLINICAL MEDICAL MANAGEMENT, INCLUDING ENTRY CRITERIA, TOXICITY MANAGEMENT, CONCOMITANT MEDICATIONS, AND CO-ENROLLMENT, THE PROTOCOL CHAIR/PROTOCOL CO-CHAIR WILL RESPOND:

- Send an e-mail message to [protocol@.tl-haartstudy.org](mailto:protocol@.tl-haartstudy.org)
- Include the protocol **number, patient identification number (**PID), and a brief relevant history.

**FOR NONCLINICAL QUESTIONS ABOUT INCLUSION/EXCLUSION CRITERIA, THE CRF SCHEDULE OF EVENTS, CASE REPORT FORMS, RANDOMIZATION/ REGISTRATION, TRANSFERS, DELINQUENCIES, AND OTHER DATA MANAGEMENT ISSUES, THE DATA MANAGER WILL RESPOND**:

- Send an e-mail message to hwong@hivnet.ubc.ca
- Include the protocol number, PID, and a detailed question.

**FOR RANDOMIZATION QUESTIONS OR PROBLEMS AND SID LISTS, THE PROTOCOL STATISTICIAN WILL RESPOND**:

- Call the Protocol Randomization Desk at (604) 806-8377 (1-800-661-4664.) and e-mail Dr. Joel Singer at joel@hivnet.ubc.ca

**FOR PROTOCOL QUESTIONS, THE PROJECT MANAGERS WILL RESPOND:**

- Send an e-mail message to Linda Apuzzo and Jennie Prasad at [protocol@.tl-haartstudy.org](mailto:protocol@.tl-haartstudy.org)

TO REQUEST COPIES OF THE PROTOCOL:

- Hard copies: Send an e-mail message to [protocol@.tl-haartstudy.org](mailto:protocol@.tl-haartstudy.org)
- Electronic copies can be downloaded from the Protocol website: www.tl-haartstudy.org

FOR SERIOUS ADVERSE EXPERIENCES (SAE) QUESTIONS:

Contact the Canadian HIV Trials Network Data Safety Monitoring Board

- Send an e-mail message to Dr. Joel Singer at joel@hivnet.ubc.ca
- Call Dr. Joel Singer at 604-806-8377 or 1-800-661-4664.

All SAEs should be reported to the Medical Monitor:

Donald Zarowny MD, MSc

Programme Head,

Scientific and Industrial Liaison,

Canadian HIV Trials Network.

Phone: (604) 806-8378

FAX: (604) 806-8005

Any phone calls must be documented by e-mail to project managers. This will be the responsibility of the site reporting the SAE.

Additional information concerning study management can be found on the protocol website.

#

# SCHEMA

**A Phase IV Randomized, Open Label Controlled Trial of Time-Limited Highly Active Antiretroviral Therapy (TL-HAART) in Patients with Acute or Early HIV Infection**

DESIGN: Within the context of a three-year phase IV randomized clinical trial, we will compare two different strategies for intervention following the diagnosis of acute or early HIV infection. Specifically, we will examine, in a randomized fashion, whether a one-year course of HAART provides lasting benefit in patients who have recently become infected with HIV. The principal study endpoint will be a comparison of the plasma viral load 24 months after initial presentation in treated vs. untreated patients. Additional endpoints will be the evolution of CD4 lymphocyte counts, safety and tolerability of antiretrovirals, and the need to initiate/re-initiate HAART over a 36 month time period following initial presentation. We will further examine whether the benefit is limited to a subset of such individuals, based on the presumed interval between exposure to HIV and initiation of therapy (acute vs. early). Staging of disease will be done in accordance with the AIEDRP staging algorithm (APPENDIX V) The highlights of the experimental design will be: 1) to attempt to include all patients meeting the NIH-approved definition of acute or early HIV disease (AIED, i.e., patients known to have been infected within the past 12 months), with emphasis on the recruitment of patients identified prior to the development of a complete anti-HIV antibody response ("pre-seroconversion"); 2) to allow for the individualization of the treatment regimen, with a special provision for patients carrying drug-resistant isolates; and 3) to administer HAART for a pre-determined length of time (i.e., one year) and then discontinue therapy. Taken as a whole, this approach will allow us to offer the opportunity to the vast majority of AIED patients to include themselves in a randomized controlled clinical trial, whose goal is to evaluate a strategy to take advantage of the potential (but not yet proven) benefits of early treatment of HIV infection while still avoiding life-long HAART. This approach will also yield generalizable data, because what is being tested is a strategy, not a specific drug regimen.

DURATION: 36 months.

SAMPLE SIZE: 180 subjects*.*

POPULATION: Patients documented to have become infected with HIV within the previous 12 months

STRATIFICATION: Acute vs. Early HIV infection (appendix V)

INTERVENTION: The only intervention will be the prescription of HAART regimens (as defined by DHHS guidelines at the time of initiation of therapy) for a period of 12 months in subjects randomized to the treatment arm of the study vs. no treatment in subjects randomized to the observational arm of the study.

## 1.0 HYPOTHESIS AND STUDY OBJECTIVES

###

### 1.1 Hypothesis

### We hypothesize that time-limited HAART (TL-HAART) given for 12 months and initiated in the first year of HIV infection will reduce the need for long-term use of HAART, and/or prolong the interval in which HAART will not be needed according to DHHS treatment guidelines for chronic HIV infection.

### 1.2 Primary Objective

To compare the steady state viral load (plasma HIV RNA concentration) in patients who have received TL-HAART to that achieved in similar patients who did not receive any antiretroviral treatment.

###

### 1.3 Secondary Objectives

1. To compare the steady state and trajectory of CD4 lymphocyte counts achieved in patients receiving TL-HAART to that achieved in similar patients who did not receive any treatment.
2. To compare the steady state and trajectory of viral load achieved in patients receiving TL-HAART in acute infection to that achieved in patients with receiving TL-HAART in early infection.
3. To compare the steady state and trajectory of CD4 lymphocyte counts achieved in patients receiving TL-HAART in acute infection to that achieved in patients with receiving TL-HAART in early infection.
4. To assess the safety and tolerability of TL-HAART in patients with acute or early HIV infection.

## 2.0 INTRODUCTION

###

### 2.1 Background

In 1996, the availability of HAART revolutionized the model of care for HIV infection. It was thought that the use of HAART could “cure” HIV infection in 3 years (2), but subsequent research has shown this model to be incorrect (3), and that long-term use of HAART is associated with significant morbidity (4). It is now felt that a well-monitored individual with chronic HIV infection and a CD4 cell count > 350 cells/mm^3^ can safely delay initiation of HAART unless plasma HIV RNA is very high (1; 5). Apart from these considerations, acute HIV infection and early HIV disease is still felt to represent a different condition from chronic HIV infection for at least three important reasons:

1) the host's immune system can be better preserved, both quantitatively and qualitatively (6)

2) the viral isolates that are transmitted may still be oligoclonal (as opposed to polyclonal in the chronic phase of the infection) and thus would be amenable to more efficient host immunologic control (7)

3) the HIV-specific immune response is still present during the first months of infection (8)

These factors, as well as some preliminary data to suggest that viral reservoirs in lymph nodes may not be fully established (9), have been presented as justification for a more aggressive therapeutic approach to acute/early HIV infection (10). Although it was initially hoped that HAART could be instituted early enough after the acquisition of HIV infection to prevent establishment of a latent viral reservoir, this reservoir appears to be established before HAART can be started (11), perhaps within 48 hours of infection (3). Current estimates are that there are about 40,000 new cases of HIV infection annually in the USA (12) and 2,100 in Canada (13). In both countries, very few cases of new infection are identified and treated in the first year of infection. A demonstration of the benefits of early treatment would be important as a means of improving the health of the infected person and as an impetus to improve methods of screening for new infections. For an individual patient treated with HAART early in HIV infection, each year of delay in the subsequent need for HAART would represent a savings of an estimated $12,000 USD drug costs and $7200 USD in other costs (14). Further, a reduction in plasma viral load set point as a result of early antiretroviral treatment could represent a major benefit with respect to secondary acquisition of HIV infection. In a recent study of HIV-infected men, multivariate analysis revealed that each log_10_ increment of plasma viral load was associated with an increase of 81% in the rate of HIV transmission from the male to the female sexual partner (15).

Smith et al (26) recently reviewed the literature on treatment of primary HIV infection. These authors, who are recognized leaders in the pathogenesis and treatment of acute and early HIV infection, concluded their review as follows:

Where does this leave the clinician who has identified a patient as acutely infected with HIV? In some regards, the question of when to treat in PHI [primary HIV infection] is similar to those patients identified with established infection; however, at the start of the disease course there is the possibility of proportionally larger benefits, making this an important question to answer. Based on the currently published data, there is no clear evidence that patients with access to antiretroviral therapy have any greater clinical benefit if therapy is introduced immediately during or prior to their seroconversion illness [67]. Despite numerous studies, none has been appropriately powered and controlled to answer this question, and we conclude that a randomized controlled trial of early short-term HAART versus deferred HAART therapy is both ethically justifiable and necessary.

It is recognized that some potential participants will have strong feelings regarding the use of HAART and may not wish to enter a protocol in which the decision to treat them will be made randomly. It is our belief that a sufficient number of people will be willing to enter a randomized trial so that our ability to accrue the desired number of participants will not be compromised.

### 2.2 Rationale

###

**2.2.1 Rationale for the strategy of not specifying HAART regimens.** The consideration of HAART in all patients with acute/early HIV infection is consistent with current DHHS guidelines for the treatment of HIV infection (16). These guidelines recommend that treatment be considered within 0-6 months of infection, but there are no data to establish either a benefit of treatment within 6 months or a lack of benefit from 6 to 12 months. At the present time, there is no consensus on the appropriate selection of initial HAART regimens for early HIV infection therefore any preferred or alternative regimen listed in the DHHS guidelines at the initiation of therapy will be allowed, and addition of a 4^th^ agent (provided not contraindicated with any of the other drugs) will also be permitted. The use of resistance testing to guide drug selection and the implementation of specific strategies to increase adherence appear to be important to ensure the success of a given regimen. When appropriate, DOT administration will be used within established programs. In all cases, selection of therapy will be optimized based on the results of genotypic resistance testing. The entry visit will take place within 14 days of screening. For those participants randomized to receive therapy, it will be started at this visit. If the results of the genotype are available at this time they will be used to determine the HAART regimen. If the results are not available treatment will be started empirically with a regimen that meets the DHHS definition of HAART which will be adjusted if necessary upon receipt of the genotype. Patients for whom no HAART regimen can be prescribed (such as would be the case for a multi-drug resistant isolate) will be excluded from the study. The use of multiple therapeutic options within each drug class is a strength of our design, allowing us to test the efficacy of a strategy (immediate vs. deferred treatment) rather than the efficacy of a single regimen. The flexibility of our design will allow us to remove HAART regimens as they are demonstrated in future clinical trials to be sub-optimal, without affecting our study design. Conversely, we will be able to add new HAART regimens as they are shown to be effective and become available in our centers. This feature may actually help us recruit patients into the trial on an ongoing basis, avoiding the problem of subjects refusing to enroll because they would not have access to a regimen they would most prefer or to the most up-to-date regimens. It will also help adherence to medications and retention of study participants.

**2.2.2 Rationale for the selection of one year as the duration for Time-Limited HAART (TL-HAART)**. TL-HAART represents a compromise to maximize the potential benefit of aggressive therapeutic intervention while minimizing the potential for a negative outcome. Most of the short-term side effects of HAART are manageable either by symptomatic treatment or drug substitution. TL-HAART avoids the long-term metabolic and morphologic toxicities of HAART. TL-HAART permits more vigorous methods (such as DOT) to maximize adherence and avoid development of drug resistance since the participants will be on therapy for only one year.. The potential benefits of the intervention (enhanced host immunity and decreased establishment of viral reservoirs and quasi-species) would all be expected to take place within the first year of therapy. The rationale for the choice of one year as the duration for TL-HAART is based on studies of the immune recovery that begins when HAART is initiated. Although circulating CD4^+^ T cell levels begin to rise very soon after this time, this is mostly due to redistribution of memory cells rather than production of new (naive) cells, which requires several months to one year to occur (24). In the MACS cohort, CD4^+^ T cell counts rose for the first two years after initiation of HAART (25). Thus, one year of therapy seems a reasonable balance between the need to suppress viremia long enough to preserve HIV-specific CD4^+^ T cells without removing the antigenic stimulus for HIV-specific CD8^+^ T cells, and allowing maximal recovery of naive CD4^+^ T cells without incurring long-term toxicities of HAART.

## 3.0 STUDY DESIGN

Within the context of a three-year randomized clinical trial, we will examine whether a one-year course of HAART provides lasting benefit in patients with acute/early HIV infection. If this is the case, we will further examine whether the benefit is limited to a subset of such individuals, based on the presumed interval between exposure to HIV and initiation of therapy. The highlights of the experimental design will be: 1) to attempt to include all patients known to have been infected within the past 12 months, with emphasis on the recruitment of patients identified prior to the development of a complete anti-HIV antibody response ("pre-seroconversion"); 2) to allow for the individualization of the treatment regimen; and 3) to administer HAART for a pre-determined length of time and then discontinue therapy.

The date of HIV infection will be estimated for all study participants according to an algorithm designed by the AIEDRP Disease Stage Working Group, which includes both Dr. Margolick and Dr. Conway. Three sets of circumstances may apply:

1. There are data to support a discrete exposure to HIV accounting for transmission of HIV. In this case, the date of this exposure will be taken as the date of infection if it has occurred 4 - 42 days before the first positive HIV plasma viral load with a negative HIV antibody test, 10 - 56 days before the first indeterminate HIV antibody test, or at least 14 days before the first positive HIV antibody test.
2. There are multiple exposures to HIV and a medical history compatible with an acute retroviral syndrome. In this case, the infection date will be estimated as 14 days prior to the onset of symptoms if the symptoms started 0 - 28 days before the first positive HIV plasma viral load with a negative HIV antibody test, 3 - 35 days before the first indeterminate HIV antibody test, or at least 7 days before the first positive HIV antibody test.
3. There is no history of a specific exposure to HIV. In this case, the infection will be dated as having occurred 21 days previously (positive HIV plasma viral load with a negative HIV antibody test), 28 days previously (positive HIV plasma viral load with an indeterminate HIV antibody test) or 85 days previously (positive HIV antibody test AND a negative detuned (less sensitive) HIV antibody test).

A Western blot will be considered negative if no bands are present, and indeterminate if some bands are present but criteria for positivity are not met. If more than one set of circumstances is met, the first will be applied.

Staging of HIV infection (acute vs. early) will be determined using the AIEDRP algorithm (Appendix V)

Taken as a whole, our approach will allow for a precise classification of individuals according to the timing of infection, enhancing our ability to test the protocol’s main hypothesis in the overall study group as well as in sub-groups representing earlier infection.

Those who agree to take part in the clinical trial and meet the inclusion criteria will be randomized either to receive TL-HAART, or to be followed off therapy. After eligibility has been established, patients will be seen within 14 days (of screening) for randomization, then at weeks 1, 2, and 4 (1 month), then every month for the next 11 months over the one-year randomization period. Thereafter, follow-up will be quarterly for the duration of the study.

For those randomized to receive therapy, the specific regimen will be selected by the study physician and the participant, according to DHHS guidelines current at the time of initiation of HAART, and modified as necessary based on the results of genotypic resistance testing conducted in real time. Early on, the evolution of anti-HIV antibodies will be measured if a fully positive Western blot test was not present at baseline. On a number of occasions, more extensive blood sampling will be done, for storage of serum, plasma, and peripheral blood mononuclear cells to test additional virologic and immunologic hypotheses that will be generated in the course of this work.

In treated patients, TL- HAART will be discontinued after 12 months of therapy, unless there is a medical contraindication to doing so, such as chronic HBV infection, CD4 cell count

< 350, or pregnancy.

**The principal study endpoint will be a comparison of the plasma viral load (plasma HIV RNA concentration) 24 months after initial presentation in treated vs. untreated patients.** Additional study endpoints will be: The plasma viral load 36 months after initial presentation in treated vs. untreated patients; the CD4 lymphocyte count 24 and 36 months after initial presentation in treated vs. untreated patients; The plasma viral load and CD4 lymphocyte count 24 and 36 months after initial presentation in patients treated in the acute vs. early stage of infection (this is the parameter that was the basis for the calculations of the study’s power); the need to initiate (or re-initiate, for those who were in the treatment arm) HAART over the period of observation in all treated vs. untreated patients; toxicity of HAART in all treated patients.

Those who decline to participate in the study or do not meet the inclusion criteria will be excluded from this study and will be given referrals to local care providers with expertise in treating HIV infection.

## 4.0 SELECTION AND ENROLLMENT OF SUBJECTS

### 4.1 Inclusion Criteria

4.1.1 HIV-1 infection acquired in the previous 12 months, as defined by one of the following:

- *Positive HIV plasma viral load and negative HIV antibody test*
- *Positive HIV plasma viral load and indeterminate HIV antibody test*
- *Positive HIV plasma viral load and positive HIV antibody test WITH:*
  - *Negative detuned HIV antibody test*
  - *OR documented negative HIV antibody test within the past 12 months*

4.1.2 No prior antiretroviral therapy (other than post-exposure prophylaxis)

4.1.3 For subjects who have been infected with HIV-1 for more than six months at study entry: HIV-1 RNA > 5,000 copies/mL obtained within 30 days of initiation of therapy performed by any laboratory that has a CLIA certification or its equivalent.

4.1.4 Laboratory values obtained within 30 days of initiation of therapy:

- Absolute neutrophil count (ANC) ≥ 750/mm^3^.
- Hemoglobin ≥ 7.0 g/dL.
- Platelet count ≥ 50,000/mm^3^.
- Creatinine ≤ 3 x upper limit of normal (ULN).
- AST (SGOT), ALT (SGPT), and alkaline phosphatase ≤ 5 × ULN.
- Total bilirubin ≤ 2.5 x ULN.

4.1.6 Negative serum pregnancy test at screening and negative urine or serum pregnancy test at the time of initiation of therapy in women of childbearing potential.

4.1.7 Age ≥ 18 years*.*

4.1.8 Ability and willingness of subject to give written informed consent.

- - 1. Ability to swallow tablets/capsules.
    2. Willingness to use barrier methods of contraception.

### 4.2 Exclusion Criteria

4.2.1 Inability to design a HAART regimen based on the results of genotypic resistance testing.

4.2.2 Two CD4+ cell count measurements <350 cells/mm^3^ obtained at least 7 days apart within 30 days of initiation of therapy obtained at laboratories that have a CLIA certification or its equivalent**.**

4.2.3 For subjects who have been infected with HIV-1 for more than six months at study entry:HIV-1 RNA < 5,000 copies/mL obtained within 30 days of initiation of therapy, measured in a laboratory that has a CLIA certification or its equivalent.

4.2.4 Pregnancy or breast-feeding.

4.2.5 Use of systemic cancer chemotherapy, systemic investigational agents, specific antiretroviral medications (with the exception of antiretroviral agents given for post-exposure prophylaxis), immunomodulators (growth factors, systemic corticosteroids, HIV vaccines, immune globulin, interleukins, interferons) within the past 30 days.

4.2.6 Active drug or alcohol use or dependence that, in the opinion of the investigator, would interfere with adherence to study requirements.

4.2.7 Serious illness requiring systemic treatment and/or hospitalization until subject either completes therapy or is clinically stable on therapy, in the opinion of the investigator, for at least 7 days prior to study entry.

4.2.8 Inability to sign informed consent.

### 4.3 Study Enrollment Procedures

4.3.1 Prior to implementation of this protocol, sites must have the protocol and consent form approved by their local institutional review board (IRB). Sites must be registered with and approved by the DAIDS Regulatory Compliance Center Protocol Registration Office.

Once a candidate for study entry has been identified, details will be carefully discussed with the subject. The subject will be asked to read and sign the consent form that was approved by the local IRB and the DAIDS Regulatory Compliance Center Protocol Registration Office.

4.3.2 The Division of AIDS has concluded that this protocol does NOT meet Federal requirements governing prisoner participation in clinical trials and should NOT be considered by local IRBs for the recruitment of prisoners. Participants who become incarcerated during the course of their participation in the study, will not be dis-enrolled, and will resume study participation at the time of their release.

4.3.3 Once it has been established that all inclusion and exclusion criteria have been met, study subjects will be randomized to receive TL-HAART or not through an automated randomization process at the Canadian HIV Trials Network, as detailed in the case report forms.

**4.4 Co-Enrollment Guidelines**

All subjects should be encouraged to enroll in the AIEDRP CORE01 Database Protocol.

## 5.0 STUDY TREATMENT (OR INTERVENTION)

5.1 Regimens, Administration, and Duration

All antiretroviral drugs to be used within this protocol will be licensed for sale for the treatment of HIV infection in Canada and the United States at the time and place at which they are used. (Table A) They will be prescribed and administered according to the version of the DHHS guidelines that are current at the time treatment is given. In Canada, all medications are 100% reimbursed by provincial drug plans. We will use once daily dosing and directly observed therapy (DOT) when appropriate. In Baltimore, most of the medications will be donated by pharmaceutical companies, or will be available free of charge from the MADAP program of the state of Maryland, or will be paid for by patient’s insurance. Study funds will be available to cover co-payments for drugs purchased through patient’s insurance. Combinations of two nucleoside/nucleotide analogues and a third or fourth agent will be used in all cases. (Note: ritonavir used for the purpose of boosting another protease inhibitor will not be considered an additional agent) As new drugs become licensed, they will be available to be used in this protocol. All patients who are randomized to receive HAART will have treatment discontinued after twelve months of therapy unless there is a medical contraindication to doing so, such as chronic HBV infection, CD4 cell count < 350 cells/mm^3^, or pregnancy. Patients who are receiving a non-nucleoside reverse transcriptase inhibitor will substitute a protease inhibitor (with or without ritonavir boosting) for 2-4 weeks prior to discontinuation of the entire regimen, if this is judged to be appropriate by the study physician.

5.2 Management of inadequate viral suppression

Virologic failure will be defined as:

1. For subjects who have achieved maximal virologic suppression (HIVRNA < 50 copies/mL), a confirmed value >50 copies/mL at any time following the 6 month visit.
2. For subjects who have not achieved maximal virologic suppression (HIVRNA < 50 copies/mL) at 6 months, a confirmed 5-fold increase from nadir (e.g. a participant whose lowest HIVRNA was < 100 but rebounds to > 500).

Participants on HAART who have viral rebound (HIVRNA >50 copies/mL) will be contacted by the study staff and questioned about adherence, diet or concomitant medications in an effort to identify factors which may have led to the rise in viremia. If two successive viral load measurements are >400 copies/mL, treatment may be modified.

The revised regimen will be selected by the study physician. All investigators, project directors and study coordinators will participate in monthly team conference calls which will be moderated by Dr. Conway. All changes in antiretroviral therapy will be discussed on the monthly call.

| **Nucleoside/Nucleotide Reverse Transcriptase Inhibitors (NRTIs)** |  |
| --- | --- |
| Abacavir | 300 mg bid (or 600 mg qd if approved by the FDA during the study period) |
| Didanosine (Videx EC^®^ formulation) | 250-400 mg qd depending on body weight^(1)^ |
| Lamivudine | 150 mg bid or 300 mg qd |
| Stavudine | 30-40 mg bid depending on body weight (80-100 mg qd of Zerit XR^®^ formulation if it becomes available during study period)^(1)^ |
| Tenofovir DF | 300 mg qd |
| Emtricitabine | 200 mg qd |
| Zidovudine | 300 mg bid |
| **Non- Nucleoside Reverse Transcriptase Inhibitors (NNRTIs)** |  |
| Efavirenz | 600 mg qd |
| Nevirapine | 200 mg bid (following 2 weeks of 200 mg qd) |
| **Protease Inhibitor (PIs)** |  |
| Amprenavir | 1200 mg bid |
| Amprenavir/Ritonavir Table A: Approved Antiretroviral Agents (June 2004) | 600/100 mg bid or 1200/200 mg qd |
| Atazanavir | 400 mg qd |
| Atazanavir/Ritonavir | 300/100 mg qd |
| Indinavir/Ritonavir | 800/100 mg bid |
| Fosamprenavir | 1400 mg bid |
| Fosamprenavir/Ritonavir | 700/100 mg bid or 1400/200 mg qd |
| Lopinavir/Ritonavir | 400/100 mg bid |
| Nelfinavir | 1250 mg bid |
| Saquinavir /Ritonavir | 1000/100 mg bid or 1600-2000/100 qd |

(1): Dose adjusted based on weight or drug interactions.

### 5.3 Product Formulation and Preparation

All products will be prescribed and used in their up-to-date commercial formulations.

### 5.4 Product Supply, Distribution, and Pharmacy

5.4.1 Study Product Acquisition

All antiretroviral agents administered in the course of the study will be obtained from commercial pharmacies and dispensed according to usual good clinical practice (GCP) guidelines.

5.4.2 Study Product Accountability

Accountability of medication dispensed by commercial pharmacies will be in accordance with local regulations.

### 5.5 Concomitant Medications

5.5.1 Required Medications

There are no required concomitant medications in this study.

5.5.2 Prohibited Medications

Use of systemic cancer chemotherapy, systemic investigational agents,

immunomodulators (growth factors, systemic corticosteroids, HIV vaccines, immune

globulin, interleukins, interferons) are prohibited throughout the course of this study. In

addition, any medications that, according to the product insert(s), cannot be

coadministered with the antiretroviral agents prescribed for a given patient are

prohibited for that patient.

5.5.3 Precautionary Medications

Any medication for which, according to the product insert(s), it is advised that precautions be taken in co-administration with the antiretroviral agents prescribed for a given patient will be precautionary for that patient.

###

### 5.6 Adherence Assessment

Adherence to HAART will be assessed by the administration of the AIEDRP adherence questionnaire (Appendix IV) at each study visit while on therapy.

## 6.0 CLINICAL AND LABORATORY EVALUATIONS

All evaluations will be performed according to the Schedule of Events (Appendix II). The definitions for the Schedule of Events included in section 6.1 define the evaluations and provide timelines, and section 6.2 includes special considerations or instructions for evaluations.

6.1.1 Prestudy evaluations

Screening

Screening evaluations to determine eligibility must be completed no more than 14 days before study randomization.

Entry

Entry evaluations must occur within 14 days of the completion of all screening evaluations. In the event that results of the genotype are not available, the patient will be randomized and if necessary his/her HAART regimen will be adjusted upon receipt of the genotype. Subjects are expected to start treatment within 24 hours of randomization. (section 6.2.3).

6.1.2 On-Study Evaluations

Study visits must be scheduled on the weeks indicated in the schedule of events with a window period of:

- 7 days on each side of target date (first year of study)
- 14 days on each side of target date (all subsequent visits)

6.1.3 Evaluations for subjects randomized to HAART who do not start study treatment.

Subjects who do not initiate study treatment within 24 hours of randomization will be reevaluated at week 1 to determine the circumstances that have led to this delay in beginning their therapy.

6.1.4 Evaluations for subjects not randomized to HAART who initiate or re-initiate study treatment

for clinical or immunologic reasons.

Subjects will be seen two weeks after initiation of therapy, and monthly until HIVRNA <50 copies/mL. After this they will follow their original visit schedule.

6.1.5 Treatment Discontinuation Evaluations

Subjects who permanently discontinue study treatment prior to completion of the one year treatment period will have all Month 12 evaluations performed as soon as possible, and then continue to be followed per the schedule of visits.

6.1.6 Post-Treatment Evaluations

Patients who will have been randomized to receive treatment in the first year of the study will continue to be followed on a quarterly basis off treatment for an additional period of two years. All untreated patients will be followed within the study protocol for a period of three years, the same duration as the treated patients.

6.1.7 Off-Study Evaluations

All participants who discontinue the study early will be contacted by telephone one month after termination. In addition, those who were receiving medication at the time of early discontinuation will be contacted again 2 months post-termination.

6.1.8 Pregnancy

### Women of childbearing potential must have a negative serum pregnancy test (β-HCG) at screening and a negative urine or serum pregnancy on the day of initiation of HAART. If a woman becomes pregnant while on study, she must be informed of her options. She will also be informed of the lack of safety data regarding the use of certain antiretroviral drugs in pregnancy. Changes of drugs within a given class or across classes will be permitted in order to maintain HAART that is safe during pregnancy. Conception after initiation of treatment will not constitute a criterion for exclusion from the study.

### 6.2 Special Instructions and Definitions of Evaluations

6.2.1 Medical History

A medical history must be present in source documents. All diagnoses should be recorded on the case report forms according to the current Adult ACTG criteria for clinical events and other diagnoses.

Any allergies to any medications and their formulations must be documented.

6.2.2 Medication History

A medication history must be present in source documents*.* All prescription and nonprescription medications as well as alternative therapies and dietary supplements taken within 30 days of entry or since the last clinic visit, as applicable, will be noted. This will include actual or estimated start and stop dates.

6.2.3 HAART

The choice of the initial HAART regimen will be clearly indicated in the source documents and in the case report forms. All modifications to HAART including initial doses, patient-initiated and/or protocol-mandated interruptions, modifications, and permanent discontinuation of treatment will be recorded on the case report forms at each study visit*.* Subject-initiated and protocol-mandated interruptions include both inadvertent and deliberate interruptions of study drug(s) dose(s) for 7 or more consecutive days.

6.2.4 Physical Exam

Complete Physical Exam

This is required at screening, and annually thereafter. It will include height at screening, and weight at screening and then again at each subsequent time point. If at any study visit should symptoms be present which warrant a physical exam, it will be performed.

Vital Signs

Temperature, pulse, and blood pressure will be collected at all visits.

Weight

This will be recorded at each study visit.

Signs and Symptoms

Any signs or symptoms that lead to a change in treatment, regardless of grade, must be recorded on the case report forms. All signs, symptoms, and toxicities must be documented in the subject’s record, but only Grade 4 signs and symptoms must be recorded on the case report forms. All HIV related signs and symptoms, HIV-related and AIDS-defining events, deaths, and toxicities must be documented. All signs, symptoms, HIV-related and AIDS-defining events, and deaths must be recorded on the case report forms within 48 hours throughout the course of the study.

Diagnoses

All confirmed and probable diagnoses made since the last visit will be recorded in the source documentation, including current status at the time of study visit. For each diagnosis, the source document must include:

1) Date of diagnosis, date of resolution

2) Method of confirmation of diagnosis or evidence for probable diagnosis

6.2.5 Adherence Questionnaires

The completion of the AIEDRP questionnaire (Appendix IV) will require an additional 5 minutes of time by the participant*.*

6.2.6 Laboratory Evaluations

Any laboratory toxicities that lead to a change in treatment, regardless of grade, must be recorded on the case report forms. At baseline, record all laboratory values. For post-baseline assessments, all laboratory values must be documented in the subject’s record but only grade 4 laboratory toxicities are to be recorded in the case report forms. Toxicities will be assessed using the Division of AIDS Table for Grading Adult Adverse Experiences,(Appendix III)

Hematology (CBC)

Hemoglobin, hematocrit, red blood cells (RBC), white blood cell count (WBC), differential WBC, absolute neutrophil count (ANC), platelets.

Chemistry Panel

Total bilirubin, AST (SGOT), ALT (SGPT), alkaline phosphatase, creatinine, indirect bilirubin, creatinine.

Lipid Panel

Total cholesterol, HDL and LDL fractionation, fasting triglycerides.

Pregnancy Test

For women with reproductive potential: Serum β-HCG or Urine test withsensitivity of 25-50 mIU/mL).

CD4 + Cell Count

Obtain absolute CD4+ cell counts and percentages at screening and at specified study visits. All laboratories performing these tests must possess a CLIA certification or equivalent. In order to perform CD4+ cell count testing that is used for patient management the laboratory also must participate in and be certified for protocol testing by the DAIDS Immunology Quality Assurance (IQA) Program, or its Canadian equivalent.

Evaluations for CD4+ cell counts should be performed at the same certified laboratory, if possible, for baseline calculation and throughout the course of the study. Because of the diurnal variation in CD4+ cell counts, determinations for individual subjects should be obtained consistently in either the morning or the afternoon throughout the study, if possible.

Note: Each time a CD4+ cell measurement is obtained, the local laboratory must perform a WBC and differential from a sample obtained at the same time.

6.2.7 Viral Load

HIV-1 RNA must be performed at screening and at subsequent study visits by a laboratory that possesses a CLIA certification or equivalent. The laboratory also must be certified by the DAIDS Virology Quality Assurance (VQA) Program or its Canadian equivalent, as the viral load determination is a primary endpoint of the study.

6.2.8 HIV EIA/WB (ELISA and Western Blot)

This will include the results of HIV antibody ELISA testing as well as Western Blot test results (done using approved assays in certified laboratories). If the Western Blot test result is not positive at the time of initial evaluation, it will be repeated at each study visit until the criteria for a positive test result are met. If the ELISA and Western Blot test results are positive at the time of initial evaluation and the diagnosis of acute/early HIV infection is in doubt, an aliquot of the initial sample will be shipped to a central laboratory for the performance of a detuned antibody ELISA assay.

6.2.9 Repository

At screening and again at months 1, 3,4,6,8,10,12 and then quarterly, expanded phlebotomy for the storage of plasma and cells will be performed. A minimum of 15 mL and a maximum of 50 mL will be collected for this purpose, to permit future studies on pertinent hypotheses of the pathophysiology of acute/early HIV infection. At other visits after month 1, 10 mL will be collected. Serum and plasma will be stored at -80^o^C in aliquots of 0.5 mL. Mononuclear cells will be cryopreserved using controlled-rate freezing and stored at -135^o^C in aliquots of 5-10 x 10^6^ cells/vial.

6.2.10 Genotype

At selected time points, a plasma sample will be taken for the performance of genotypic drug resistance testing in local laboratories. Samples that cannot be processed in this manner will be sent to the academic laboratory of Dr. Brian Conway at the University of British Columbia, where they will be analyzed using the TruGene Assay (Bayer Diagnostics, Mississauga, ON, Canada).

### 6.3 Off-Drug Requirements

### Additional safety monitoring and reporting of serious adverse experiences (SAEs) continue to be required upon completion or discontinuation of study treatment regardless of whether a protocol follow-up period is scheduled to occur. As specified in the current Division of AIDS *Serious Adverse Experience (SAE) Reporting Manual,* adverse experiences occurring during the immediate 8-week period after the last dose of study treatment which meet SAE reporting requirements must be reported to the DAIDS according to the SAE reporting procedures listed in section 11.4.

## 7.0 TOXICITY MANAGEMENT

##

For patients randomized to receive treatment, the HAART regimen will be selected or adjusted based on the results of the genotypic resistance test done at the time of screening. Selection and/or adjustment will be done by the study physician, who will then inform the chair or co-chair (in the case when neither the chair nor the co-chair is the treating physician) of the choice. The standard ACTG/CTN approach will be used to grade and address drug toxicity on an ongoing basis. There will be no protocol-mandated criteria for a substitution or discontinuation of HAART, such decisions being left to the standards of clinical practice, with all drug-associated toxicities being documented. Drug substitutions within the same class of drug (NRTI for NRTI, NNRTI for NNRTI or PI for PI) will be preferred, as decided by the study physician, but changes across classes will also be permitted. If the chair or co-chair of the protocol is not the treating physician, they will be informed of all decisions to change or discontinue HAART regimens on the monthly conference calls.

## 8.0 CRITERIA FOR TREATMENT DISCONTINUATION

- Drug-related toxicity (see section 7.0 Toxicity Management).
- Development of medical condition which makes it necessary to temporarily or permanently discontinue medication in the opinion of the study investigator.
- Requirement for prohibited concomitant medications (see section 5.4)
- Failure by the subject to attend three consecutive monthly visits.
- Subject repeatedly noncompliant with study medications as prescribed.
- Request by the subject to withdraw.
- Request of the primary care provider if s/he thinks the study is no longer in the best interest of the subject.
- Clinical reasons believed life threatening by the study physician, even if not addressed in the toxicity management of the protocol.
- Subject judged by the investigator to be at significant risk of failing to comply with the provisions of the protocol as to cause harm to self or seriously interfere with the validity of study results.
- Subject reaches a defined study endpoint, if applicable.
- At the discretion of the NIAID, CTN, investigator, or other regulatory authority.

## 9.0 STATISTICAL CONSIDERATIONS

Any patient who is randomized will be evaluated. Parametric and distribution-free statistical methods will be used to analyze quantitative and qualitative/categorical data. Within group comparisons of baseline and follow-up measurements of plasma viral load (log-transformed) and CD4 lymphocyte counts will be conducted using Student’s paired t-test. For the principal study endpoint, a comparison of the mean log-transformed plasma viral load measures 24 months after the initial presentation in all treated vs. untreated subjects will be carried out using Student’s unpaired t-test and repeated measures analysis of variance. A similar approach will be taken to the comparison of plasma viral load measures at other time points or between sub-groups. Absolute CD4 lymphocyte counts will be treated in the same way. For issues of immunologic disease progression (defined as CD4 cell count < 350 cells/mm^3,^, and requiring a consideration of HAART), survival analytic methods (e.g. Kaplan-Meier, Cox proportional hazard regression) will be used. For other data (such as drug toxicity and treatment discontinuation), descriptive statistics will be used, with appropriate comparisons between groups as indicated.

### 9.1 General Design Issues

###

### 9.2 Endpoints

9.2.1 Primary Endpoints

- Comparison of the plasma viral load 24 months after initial presentation in all treated vs. untreated patients.

9.2.2 Secondary Endpoints

- Comparison of the plasma viral load 36 months after initial presentation in all treated vs. untreated patients.
- Comparison of the CD4 lymphocyte count 24 and 36 months after initial presentation in all treated vs. untreated patients.
- Comparison of the plasma viral load 24 and 36 months after initial presentation in patients treated in the acute vs. early stage of infection.
- Comparison of the CD4 lymphocyte count 24 and 36 months after initial presentation in patients treated in the acute vs. early stage of infection.
- Toxicity of HAART in all treated patients.

### 9.3 Randomization and Stratification

### 9.4 Sample Size and Accrual

###

Viral load set points will be evaluated at 12 and 24 months after completion of the 12 months randomization period, i.e., 24 and 36 months after entry into the study. For each patient, we observe the viral load at the pre-defined times and compare the treated and untreated groups. Based on preliminary studies at Weill Cornell School of Medicine in which the mean viral load set point after stopping HAART was 4.2 log_10_ copies/ml with SD = 0.5 log_10_ copies/mL, we anticipate a standard deviation of 0.5 log_10_ copies/mL for the plasma viral load at the times this endpoint will be measured. This is illustrated in the sample size tables below. Further, based on data from the study by Mellors et al from the MACS, which suggest that a difference of 0.25 log_10_ copies/mL may confer a meaningful long-term clinical benefit, we have evaluated our expected power to detect a difference of this magnitude. Based on these assumptions, we can calculate the number of patients/group that would be required to achieve a given power for detecting a specific measured virologic benefit of TL-HAART:

| Virologic Benefit of TL-HAART | N/group for β=0.80 | N/group for β=0.90 |
| --- | --- | --- |
| 0.25 log_10_ copies/mL | 64 (93, 144)^*^ | 84 (121,189) |
| 0.50 log_10_ copies/mL | 16 (24,36) | 21 (31,48) |
| 0.75 log_10_ copies/mL | 7 (11,16) | 10 (14,21) |

* Power assuming a standard deviation of 0.5 (0.6, 0.75) log_10_ copies/ml of HIV RNA, and α=0.05.

Thus, with our projected enrollment of 180 subjects, we would have 90 subjects per group which would give us more than 90% power to detect a benefit as small as 0.25 log_10_ copies/mL. If the difference is only slightly greater, we will be able to detect it with β > 0.95. If need be, the sample size could be increased by extending the recruitment period to 30 months rather than 24. If we more conservatively estimate that the standard deviation of the observations of viral load set points will be somewhat larger, e.g., 0.6 or 0.75 log_10_ copies/mL of HIV RNA (as observed by Gallant et al (27) over a longer time period with a more heterogeneous group than we will study), rather than 0.5, then the sample size may still be large enough to detect a difference of 0.25 log_10_ copies/ml of HIV RNA with a power of 0.8. Some would argue that the greatest (and most significant) differences will be present in subjects infected most recently (acute) . It is our intention to recruit 60 such subjects (30/group) in addition to the 120 patients enrolled in the early stage of infection. With this recruitment, the following table shows the power of the study as a function of the expected virologic benefit:

| Virologic Benefits of TL-HAART | Infected 0-2 mo (30/group) | Infected 2-12 mo (60/group) | Infected 0-12 mo (60/group) |
| --- | --- | --- | --- |
| 0.25 log_10_ copies/mL | 35% (26,18)* | 61% (46,32) | 78% (63,45) |
| 0.50 log_10_ copies/mL | 89% (75,56) | 99% (96,85) | > 99% (99,95) |
| 0.75 log_10_ copies/mL | > 99% (98,89) | > 99% (>99,99) | > 99% (>99,99) |

* Power assuming a standard deviation of 0.5 (0.6, 0. 75) log_10_ copies/ml of HIV RNA, and α=0.05.

If the standard deviation of the viral load set points is 0.5 log_10_ copies/mL, the study is more than adequately powered to detect a difference of 0.25 log_10_ copies/mL unless only a relatively small benefit of treatment is observed only in the acute sub-group. . If the standard deviation is larger, the ability to find a difference of 0.25 log_10_ copies/mL is reduced for this sub-group, but remains high for larger treatment effects. If there is substantial variation in disease progression across individuals, the power will be lower than that given in the table and larger sample sizes would be needed. This is because the variability of the changes includes not only the measurement error associated with the viral load assay but also natural variation in disease progression across individuals (irrespective of whether they are on or off treatment) and/or a differential effect of treatment across individuals. If this were true, the assumed standard deviation of plasma viral load of 0.5 log_10_ copies/mL would be an underestimate. However, it is extremely unlikely that this situation pertains. The standard deviations on which we have based our calculations were observed in real studies in which HAART was discontinued and patients were followed. Therefore, they take into account at least some of the variability in disease course that may pertain to the situation of our study. For this reason, we are quite confident that the protocol we have put forward is feasible and will address the issue for which it is designed.

**Patient Recruitment** - All patients in whom a diagnosis of AIED can be made will be offered the opportunity to participate in the study. Over two years, we will recruit 180 patients into the protocol, including significant numbers of IDUs, including 48 in Baltimore and 132 at Canadian sites (overall, 1-2/week in all the sites combined).

**Recruitment at Canadian sites -** In order to enhance recruitment, we have developed centralized clinical infrastructures in Vancouver. This serves to facilitate outreach efforts for subject enrolment with an emphasis on IDUs and women who do not easily access health care (including Aboriginal women), provide community-based nursing and laboratory support for health care centers and community clinics that wish to enroll subjects and co-ordinate their follow up. *Providence Health Care:* This is the tertiary care hospital center at which all of the participating Vancouver physicians have admitting privileges. It houses a dedicated 18-bed HIV ward. The emergency room is the preferred point of initial contact for IDUs in the vicinity, who may not yet be known to be infected with HIV. It is accepted practice to perform HIV testing on these individuals on a routine basis. **In the past 4 years, a mean of 8 patients/year has been referred to our AIED program**. *Community Physicians/AIDS Service Organizations:* They have been targeted by presentations on AIED at local meetings, and distribution of a postcard in prominent community locations. This system is **expected to continue to yield a mean of 16 patients/year**. *Provincial Reference Laboratory:* This laboratory, located in Vancouver, performs all HIV antibody testing for the province of British Columbia. There are 450 new positive tests every year. About 30-40 patients/year test positive and do not have a family physician and may be referred into our study. **We expect 6 referrals/year**. *Vancouver Coastal Health Authority:* Dr. Conway is the Consultant in Infectious Diseases Specialist based at the Pender Community Health Center, which provides care for 200 HIV-infected individuals, including 6 new referrals/month. This clinic, which caters mostly to IDUs, is also linked to three other clinics in the area, collectively providing care to another 200 HIV-infected individuals, including 3-5 new referrals/month: a) Three Pillars Clinic (Dr. Horvath); b) Portland Hotel Clinic (Dr. Mate); and c) Yaletown Clinic (Dr. Jiwa). **This network is expected to yield 12 AIED patients/year**. We will keep in contact with private physicians who have large HIV patient bases and who regularly refer patients to us at regular intervals. We will also provide feedback to each physician source. **This is expected to yield 8 patients/year**. After major conferences, Dr. Conway is often the invited speaker at HIV Update dinner meetings which are very well attended by the local HIV medical community. This will represent a unique opportunity to publicize findings in the field of AIED and enhance the study’s visibility. This is **expected to yield 6 patients/year**. **Our projection of 50 new AIED patients/year in Vancouver is entirely realistic**. *Cool Aid Community Health Centre, Victoria:* This is a community and referral clinic in downtown Victoria (active census 200 patients), directed by Dr. Chris Fraser, a HIV/addiction specialist. **It is expected to generate 8 referrals/year**. *Sunnybrook Hospital HIV Clinic, Toronto:* This is a tertiary care HIV referral clinic in Toronto, under the direction of Dr. Anita Rachlis. **It is expected to generate 10 new referrals/year**. *Clinique Médicale du Quartier Latin, Montreal:* This is a large HIV clinic in Montreal, with over 2,000 patients. Dr. Pierre Coté is its research director, and the clinic has a total of three full time research coordinators in addition to outreach workers. Over the past two years, it has been active within the Canadian portion of the AIEDRP network and has enrolled 37 patients (30 men, 7 women). **We project a continued enrollment of 12 patients/year.**

**Recruitment in Baltimore -** *ALIVE I/II Cohort Study*. These are NIDA-funded cohort studies of the natural history of HIV infection in IDUs (ALIVE I) and risk factors for infection with HIV among seronegative IDUs (ALIVE II). This population is almost entirely African American. All HIV negative participants in these studies are screened every 6 months for HIV infection. ALIVE II (S. Mehta), principal investigator) has received funding through 2008. *The Johns Hopkins Hospital HIV Service*. The Moore clinic follows 3100 HIV infected people, mostly African American (78%) and male (67%). *Chase-Brexton Health Services (CBHS) -* This large multi-disciplinary clinic is located in mid-town Baltimore city and provides HIV and other primary care services to over 1,100 patients; *Baltimore City Health Department Sexually Transmitted Disease (STD) Clinics* - The Johns Hopkins Infectious Diseases Division provides professional staff for the clinical operation of the STD clinics. Approximately 30,000 patient-visits occur annually in two city STD clinics in which the HIV seroprevalence is 5.2%. Other sources will include *Local Hospital inpatient wards; Community Physicians and Clinics;* *Clinic Based Recruitment.* We will also provide feedback to each physician source. After major HIV Conferences and Dinner meetings outside of Baltimore, there are several HIV Update dinner meetings, which are very well attended by the local HIV medical community. **Based on the track record of these sources, we anticipate enrolling 24 patients/year into the study.**

**Strategies for Retention –** In keeping with current practices, we will pay $15 (Baltimore) or $8 (Canada) per study visit. Participants will receive an appointment card at each visit, which includes telephone numbers and pager numbers of study staff. Locator forms will be obtained at baseline and updated every 3 months. Attempts to reschedule missed visits are made immediately with subsequent follow-up by a social worker, pharmacist or other relevant health care professional. We will also search hospital and correctional institution databases for recent admissions. We would also consider off-site visits (e.g. to participant’s homes, outside clinics). The study staff is devoted to services that will encourage continued participation by the participant providing flexibility in scheduling (e.g., evening visits), providing basic counseling and referral services as required, coordinating appointments and blood draws with their medical providers, and being available to discuss the many concerns that surface upon receiving a new HIV diagnosis. Referrals for professional counseling and support are provided as needed, and review of laboratory results and their significance occurs at each visit.

###

### 9.5 Monitoring

The Data Safety and Monitoring Committee of the Canadian Trials Network will serve as the DSMB for this study. The study will be reviewed every six months.

HAART associated toxicity will be assessed as follows:

- Toxicity leading to treatment discontinuation
- Evaluation of Serious Adverse Events
- Evaluation of grade 3 and 4 Adverse Events

Viral load and T-cell data in the treated versus untreated groups will be compared.

### 9.6 Analyses

Primary Endpoint

 The 24 month log plasma viral load will be compared between allocated groups on an intent-to-treat basis using linear regression to adjust for baseline plasma viral load and CD4 lymphocyte count.  All patients who have at least one post baseline visit will be included in the analysis. Patients who withdraw from follow-up prior to 24 months will have their 24 month viral load estimated using regression techniques based on group and individual trajectories.  Secondary sensitivity analyses using other analytic strategies including last observation carried forward will be used to assess the robustness of the result.

 Secondary Endpoints

Between group comparison of log plasma viral load at 36 months, and CD4 lymphocyte count at 24 and 36 months will be conducted in the same way as the analysis of 24 month viral load.

Subgroup analyses comparing treatment groups on the CD4 lymphocyte count and plasma viral load outcomes at 24 and 36 months will be carried out similarly, focusing (a) on patients treated in the acute stage and (b) patients treated early.

 A further analysis for each of the endpoints will be done using baseline CD4 lymphocyte count and plasma viral load, stage of disease, the interaction of stage and treatment, and treatment as predictors of outcome in a linear regression analysis.  This additional analysis is being done to perform formal statistical assessment of whether there are treatment by stage interactions. (The presence of an effect in one subgroup, and the lack thereof in another does not necessarily imply that the difference between the result in the two strata is statistically significant).

The toxicity of HAART will be presented using descriptive statistics as follows:

(a) percentage of patients discontinuing therapy because of toxicity

(b) percentage of patients suffering a serious adverse event (SAE)

(c) percentage of patients suffering a grade 3 or 4 adverse reaction

 All adverse events, including minor adverse events will be classified using the ACTG template.

 Confidence intervals around the percentages will be calculated.

##

## 10.0 PHARMACOLOGY PLAN

*NOT APPLICABLE*

###

### 10.1 Pharmacology Objectives

###

### 10.2 Pharmacology Study Design

###

### 10.3 Primary and Secondary Data, Modeling, and Data Analysis

###

### 10.4 Anticipated Outcomes

## 11.0 DATA COLLECTION AND MONITORING AND ADVERSE EXPERIENCE REPORTING

### 11.1 Records to Be Kept

Case report forms will be provided for each subject. Subjects must not be identified by name on any case report forms. Subjects will be identified by the patient identification number (PID) and study identification number (SID) provided by the Canadian HIV Trials Network Data Management Center upon randomization.

### 11.2 Role of Data Management

11.2.1 Instructions concerning the recording of study data on case report forms will be provided by the Canadian HIV Trials Network Data Management Center. Each study site is responsible for keying the data in a timely fashion.

11.2.2 It is the responsibility of the Canadian HIV Trials Network Data Management Center to assure the quality of computerized data for this study. This role extends from protocol development to generation of the final study databases.

###

### 11.3 Clinical Site Monitoring and Record Availability

11.3.1 Site monitors under contract to the National Institute of Allergy and Infectious Diseases (NIAID) will visit participating clinical research sites to review the individual subject records, including consent forms, case report forms, supporting data, laboratory specimen records, and medical records (physicians’ progress notes, nurses’ notes, individuals’ hospital charts), to ensure protection of study subjects, compliance with the protocol, and accuracy and completeness of records. The monitors also will inspect sites’ regulatory files to ensure that regulatory requirements are being followed.

11.3.2 The investigator will make study documents (e.g., consent forms, drug distribution forms, case report forms) and pertinent hospital or clinic records readily available for inspection by the local IRB, the site monitors, the NIAID, the Office for Human Research Protections (OHRP), or other regulatory authorities for confirmation of the study data.

### 11.4 Serious Adverse Experience (SAE) Reporting

11.41 Serious adverse experiences must be documented on the Division of AIDS Serious Adverse Experience (SAE) Reporting Form and submitted to DAIDS through the Regulatory Compliance Center (RCC) and are defined in the current DAIDS *Serious Adverse Experience (SAE) Reporting Manual.* For SAE reporting of laboratories or medical conditions not found on the DAIDS table for Grading Adult Adverse Experiences (Appendix III), refer to “estimating severity grades scale” found in the SAE section of the DAIDS Serious Adverse Experience (SAE) Reporting Manual.

11.42 Participating sites will be provided with the following materials:

- DAIDS Policy for SAE Reporting on Non-IND Studies
- DAIDS SAE Reporting Manual
- DAIDS Table for Grading Severity of Adult Adverse Experiences
- DAIDS Serious Adverse Events (SAE) Form

11.43 Sites must also adhere to local regulatory agency (IRB) requirements for serious adverse event  reporting. Specifically, for Baltimore, all SAEs will be reported to the Johns Hopkins Bloomberg School of Public Health Committee on Human Research, and Canadian sites will notify their local IRBs. Any safety reports submitted by sites to the local regulatory authority must be kept in the essential documents file. These reports must be submitted to the DAIDS Clinical Representative.

11.44 All SAEs will be reported to the medical monitor for this study within 3 working days of notification of the event. The medical monitor for this study is:

Donald Zarowny MD, MSc

Phone: (604) 806-8378

FAX: (604) 806-8005

## 12.0 HUMAN SUBJECTS

###

### 12.1 Institutional Review Board (IRB) Review and Informed Consent

This protocol and the informed consent document (Appendix I) and any subsequent modifications will be reviewed and approved by the IRB or ethics committee responsible for oversight of the study. A signed consent form will be obtained from the subject. The consent form will describe the purpose of the study, the procedures to be followed, and the risks and benefits of participation. A copy of the consent form will be given to the subject, and this fact will be documented in the subject’s record.

### 12.2 Subject Confidentiality

All laboratory specimens, evaluation forms, reports, and other records that leave the site will be identified by coded number only to maintain subject confidentiality. **All records will be kept locked.** All computer entry and networking programs will be done with coded numbers only. Clinical information will not be released without written permission of the subject, except as necessary for monitoring by IRB, the NIAID, the OHRP, or other regulatory authority.

### 12.3 Study Discontinuation

The study may be discontinued at any time by the IRB, the NIAID, or other government agencies as part of their duties to ensure that research subjects are protected.

#

## 13.0 PUBLICATION OF RESEARCH FINDINGS

Publication of the results of this trial will be governed by the policies of the Acute Infection Early Disease Research Program (AIEDRP), of which both Dr. Margolick and Dr. Conway (chair and co-chair of this protocol) are members of the executive committee.

## 14.0 BIOHAZARD CONTAINMENT

As the transmission of HIV and other blood-borne pathogens can occur through contact with contaminated needles, blood, and blood products, appropriate blood and secretion precautions will be employed by all personnel in the drawing of blood and shipping and handling of all specimens for this study, as currently recommended by the Centers for Disease Control and Prevention and the National Institutes of Health.

All infectious specimens will be transported using packaging mandated in the Code of Federal Regulations, 42 CFR Part 72. Please also refer to individual carrier guidelines, e.g., Federal Express, Airborne Express, for specific instructions.

## 15.0 REFERENCES

1. Yeni,P.G., Hammer,S.M., Carpenter,C.C.J., Cooper,D.A., Fischl,M.A., Gatell,J.M., Gazzard,B.G., Hirsch,M.S., Jacobsen,D.M., Katzenstein,D.A., Montaner,J.S., Richman,D.D., Saag,M.S., Schechter,M., Schooley,R.T., Thompson,M.A., Vella,S., and Volberding,P.A., Antiretroviral therapy for adult HIV infection in 2002: updated recommendations of the International AIDS Society-USA Panel. *JAMA* **288**, 222-235, 2002.

2. Perelson,A.S., Essunger,P., Cao,Y., Vesanen,M., Hurley,A., and Saksela,K., Decay characteristics of HIV-1-infected compartments during combination therapy. *Nature* **387**, 188-191, 1997.

3. Finzi,D., Blankson,J., Siliciano,J.D., Margolick,J.B., Chadwick,K.R., Pierson,T., Smith,K., Lisziewicz,J., Lori,F., Flexner,C., Quinn,T.C., Chaisson,R.E., Rosenberg,E., Walker,B., Gange,S.J., Gallant,J., and Siliciano,R.F., Latent infection of CD4^+^ T-cells provides a mechanism for lifelong persistence of HIV-1, even in patients on effective combination therapy. *Nature Medicine* **5**, 512-517, 1999.

4. Carr,A. and Cooper,D.A., Adverse effects of antiretroviral therapy. *Lancet* **356**, 1423-1430, 2000.

5. Phair,J.P., Mellors,J.W., Detels,R., Margolick,J.B., and Munoz,A., Virologic and immunologic values allowing safe deferral of antiretroviral therapy. *AIDS* **16**, 2455-2459, 2002.

6. Soudeyns,H., Campi,G., Rizzardi,G.P., Lenge,C., Demarest,J.F., Tambussi,G., Lazzarin,A., Kaufmann,D., Casorati,G., Corey,L., and Pantaleo,G., Initiation of antiretroviral therapy during primary HIV-1 infection induces rapid stabilization of the T-cell receptor beta chain repertoire and reduces the level of T-cell oligoclonality. *Blood*  **95**, 1743-1751, 2000.

7. Karlsson,A.C., Birk,M., Lindback,S., Gaines,H., Mittler,J.E., and Sonnerborg,A., Initiation of therapy during primary HIV type 1 infection results in a continuous decay of proviral DNA and a highly restricted viral evolution. *AIDS Res.Hum.Retroviruses* **17**, 409-416, 2001.

8. Rosenberg,E.S., Altfeld,M., Poon,S.H., Phillips,M.N., Wilkes,B.M., Eldridge,R.L., Robbins,G.K., D'Aquila,R.T., Goulder,P.J.R., and Walker,B.D., Immune control of HIV-1 after early treatment of acute infection. *Nature* **407**, 523-526, 2000.

9. Pantaleo,G., Cohen,O.J., Schacker,T., Vaccarezza,M., Graziosi,C., Rizzardi,G.P., Kahn,J., Fox,C.H., Schnittman,S.M., Schwartz,D.H., Corey,L., and Fauci,A.S., Evolutionary pattern of human immunodeficiency virus (HIV) replication and distribution in lymph nodes following primary infection: implications for antiviral therapy. *Nat Med* **4**, 341-345, 1998.

10. Panel on Clinical Practices for Treatment of HIV Infection, Guidelines for the use of antiretroviral agents in HIV-infected adults and adolescents. *www.hivatis.org* 2002.

11. Chun,T.W., Engel,D., Berrey,M.M., Shea,T., Corey,L., and Fauci,A.S., Early establishment of a pool of latently infected, resting CD4(+) T cells during primary HIV-1 infection. *Proc.Natl.Acad.Sci.U.S.A* **95**, 8869-8873, 1998.

12. Glendening,P.N., Townsend,K.K., Benjamin,G.C., and Solomon,L., "The 1999 Maryland Annual HIV/AIDS Report," pp 36-102, 1999.

13. Health Canada Report. *Health Canada Report* 2002.

14. The HIV Research Network, Hospital and outpatient health services utilization among HIV-infected patients in care in 1999. *J Acquir.Immune Defic.Syndr.* **30**, 21-26, 2002.

15. Tovanabutra,S., Robison,V., Wongtrakul,J., Sennum,S., Suriyanon,V., Kingkeow,D., Kawichai,S., Tanan,P., Duerr,A., and Nelson,K.E., Male viral load and heterosexual transmission of HIV-1 subtype E in northern Thailand. *J Acquir.Immune Defic.Syndr.* **29**, 275-283, 2002.

16. Guidelines for the Use of Antiretroviral Agents in HIV-1-Infected Adults and Adolescents. Available at [http://AIDSinfo.nih.gov](http://aidsinfo.nih.gov/).  Accessed March 24, 2004.

17. Robbins GK, De Gruttola V, Shafer RW, et al. Comparison of sequential three-drug regimens as initial therapy for HIV-1 infection. N Engl J Med 349:2293-2303, 2003.

18. Conway B, Prasad J, Reynolds R, et al. Directly observed therapy for the management of HIV-infected patients in a methadone program. Clin Infect Dis, in press, 2004.

19. Pecora Fulco P, Kirian MA. Effect of tenofovir on didanosine absorption in patients with HIV. Ann Pharmacother 37:1325-1328, 2003.

20. Pozniak AL, Gallant JE, Staszewski S, et al. Similar 96-Week Efficacy Profile Regardless of Baseline Characteristic Variable for Tenofovir Disoproxil Fumarate (TDF) Versus Stavudine (d4T) When Used in Combination With Lamivudine and Efavirenz in Antiretroviral-Naive Patients. Poster 559, 2nd International AIDS Society Conference on HIV Pathogenesis and Treatment, 2003.

21. Nunez, M., Rodriguez-Rosado, R., Soriano, V., and Gonzalez-Lahoz, J. M. The SENC trial: Spanish efavirenz vs nevirapine comparison trial. Preliminary results of a prospective, randomized, controlled, open-label study in HIV^+^ naive individuals. ICAAC, 2000, Abstract 472 . 2000.

22. van Leth F, Phanuphak P, Ruxrungtham K, et al. Comparison of first-line antiretroviral therapy with regimens including nevirapine, efavirenz, or both drugs, plus stavudine and lamivudine: a randomised open-label trial, the 2NN Study. Lancet 363:1253-1263, 2004.

23. Squires KE, Thiry A, Giordano M, for the AI424-034 International Study Team. Atazanavir (ATV(+/-RTV)) QD and efavirenz (EFV) QD with fixed-dose ZDV+3TC: Comparison of antiviral efficacy and safety through wk 24 (AI424-034). Program and abstracts of the 42nd Interscience Congress on Antimicrobial Agents and Chemotherapy, 2002; San Diego, California. Abstract H-1076.

24. Autran,B., Carcelain,G., Li,S., Blanc,C., Mathez,D., Tubiana,R., Katlama,C., Debre,P., and Leibowitch,J., Positive effects of combined antiretroviral therapy on CD4^+^ T cell homeostasis and function in advanced HIV disease. *Science* **277**, 112-116, 1997.

25. Tarwater,P.M., Margolick,J.B., Jin,J., Phair,J.P., Detels,R., Rinaldo,C., Giorgi,J., and Munoz,A., Increase and plateau of CD4 T-cell counts in the 3(1/2) years after initiation of potent antiretroviral therapy. *J Acquir Immune Defic Syndr* **27**, 168-175, 2001.

26. Smith, D. E., Walker, B.D., Cooper, D.A., Rosenberg. E.S., Kaldor, J.M., Is antiretroviral treatment of primary HIV infection clinically justified on the basis of current evidence? *AIDS*, March 2004; 18:709-18

27. Tarwater PM, Parish M, Gallant JE, Prolonged treatment interruption after immunologic response to highly active antiretroviral therapy. *Clin Infect Dis* (United States), Dec 2003, 37(11) p1541-8

APPENDIX I

**SAMPLE CONSENT FORM**

| **Title of Research Project:** A Randomized Trial of HAART in Acute/Early HIV Infection |
| --- |

**Principal Investigator:**

**Introduction:**

This consent form explains the research study you are being asked to join. Please review this form carefully and ask any questions about the study before you agree to join. You may ask questions at any time after joining the study.

**Purpose of Research Project:**

You are being asked to take part in this study because you have been infected with HIV, possibly within the past year. HIV is the virus that causes AIDS. HIV can hurt your body’s ability to fight infection (immune system). This happens when HIV lowers the number of infection-fighting cells (T-cells) in your blood. We know a lot about how to treat HIV in people who have had the virus for a long time. However, we still do not know the best way to treat people who have been infected within the past year.

When people have had HIV for a long time and have less T-cells than they should, they usually start treatment. There are many medicines approved to treat HIV. People usually take a combination of 3 – 4 medicines. Once these medicines are started, they are usually taken for many years. This is known as HAART (Highly Active Antiretroviral Therapy).

We think that if we give HAART for 12 months during the first year of HIV infection, and then stop the treatment, we might be able to keep your immune system working better and increase the number of years that you can go before you need to take HAART again. We do not know this for sure; and this is what we hope to learn through this study.

We are also going to study the side effects that the people taking HAART develop. We want to see if taking HAART early and only for one year leads to fewer side effects.

In order to study these questions, we will give treatment to half the people who are in this study, and half will not receive treatment. Who will receive treatment will be decided by chance. The treatment will last one year. We will then follow both the people who received treatment and those who did not. We want to see if giving treatment right away is more helpful than waiting to start treatment.

According to the current guidelines for the treatment of HIV infection, the benefits of treating people during the acute phase are based on theory and have not been proven in studies. We hope to answer this question through this study.

This study will be done at six centers throughout Canada and the United States. We plan to enroll 180 people into this study. Your participation in the study will last for 3 years.

**Procedures:**

**The Screening Visit:**

Before you can join this study, you will have a screening visit to find out if you can be in this study. You will have standard tests used in the care of patients with HIV.

At the screening visit we will:

- Ask you questions about your medical history.
- Ask how you think you were exposed to HIV.
- Ask you about any medications that you are taking.
- Talk about high-risk behaviors (like using drugs or having sex without a condom).
- Draw blood for routine laboratory tests. The total blood drawn at this visit will be about 6 tablespoons and will look at your red and white blood cells (hematology), your liver and kidney (chemistry) and the amount of fats in your blood (lipids).
- Measure your T-cell count, which tell us how well your immune system is working.
- Measure the amount of HIV in your blood, (viral load).
- Test for HIV antibodies (tests which tell us you are HIV positive).
- Test your HIV drug sensitivity (genotype). This means we will find out which drugs are likely to work against your HIV infection.
- Test for Hepatitis B and Hepatitis C (diseases which can affect your liver) and syphilis.
- If you are a woman who can have a child, you will have a pregnancy test.

If these tests tell us that you were exposed to HIV infection more than 12 months ago, you will not be able to take part in this study. In that case, we will help you find a doctor who can treat your HIV infection.

If the tests show that you can be in this study, you will be invited to come back to join the study.

**The Randomization Visit:**

If you want to take part in this study, you will come back for your next study visit within fourteen days. At this visit you will have a complete physical exam. You will be randomized (like flipping a coin). This means you will either receive Highly Active Antiretroviral Therapy (HAART) right away or you will not be treated unless it is clinically needed. Randomization will be done by telephone to a computerized randomization center while you are with the study coordinator. You will find out then if you will be getting treatment.

If you are going to receive treatment, a study doctor will review your HIV sensitivity test. This doctor will recommend medicines that should work well for you. This will be based on what we know about treating HIV in people who have been infected for more than one year. We will talk about these drugs with you and ask you questions about your lifestyle. We will ask you about the kinds of foods you eat and how early you get up in the morning. We want to be sure that we have chosen a combination of drugs that you will be able to take. We will tell you about the side effects of each drug, how often you will need to take it, and how it should be taken.

There are many medicines available to treat HIV. If you cannot take a particular drug, we can switch you to another one that will be easier for you to take. It is very important that you take your medicines the way that they are prescribed. It is important that you tell the study staff if you are having problems with your medicines (like side effects or missing doses).

We will draw blood at this visit, for hematology, chemistry, t-cells and viral load tests. We may repeat an HIV test. We will draw blood to store for future testing. The total blood drawn at the randomization visit will not be more than about 6 tablespoons.

If you are a woman who can have a baby and are randomized to begin treatment, you will have another pregnancy test.

**Additional Study Visits:**

Additional study visits will take place after 1 and 2 weeks. Then you will come back once a month for 1 year. At each of these visits, we will ask you questions about your health, any medications that you are taking (and any side effects you may be having), and high-risk behaviors. If you are taking antiretrovirals (medicines to treat HIV) we will ask you to complete a questionnaire about how you are doing with them. Blood will be drawn and a physical exam will be done according to the schedule below.

Weeks 1, 2

- Blood for hematology and chemistry (about 2 tablespoons)
- Blood to test for HIV antibodies (one teaspoon, only if your test was not positive at the last visit)
- Physical exam (only if you are having any symptoms)

Months 1, 2, 4, 8, 10, 11

- Blood for hematology and chemistry and viral load (about 3 tablespoons)
- Blood to test for HIV antibodies (one teaspoon, only if your test was not positive at the last visit)
- Physical exam (only if you are having any symptoms)

Months 3, 6, 9 and every three months until the end of the study

- Blood for hematology and chemistry, viral load, T-cells and storage for future testing (about 6 tablespoons)
- Physical exam (only if you are having any symptoms)

Month 12 and every twelve months until the end of the study

- Blood for hematology and chemistry, viral load, T-cells, lipids and storage for future testing (about 6 tablespoons)
- Physical exam

After month twelve, study medicine will be stopped in all people who have been getting treatment. Treatment will not be stopped if there are medical reasons why treatment should be continued. Those medical reasons include pregnancy, low T-cells or chronic hepatitis B infection. If you have been getting treatment you will come in for visits 1, 2 and 4 weeks after stopping treatment. We will draw blood for viral load and storage for future testing at these visits.

Everyone taking part in this study will come in every 3 months until the end of the study (month 36). At each visit we will ask you about how you are doing and blood will be drawn.

Each study visit should take about 30 minutes. The first two visits may take up to 1 hour. The total amount of blood drawn at each visit will be different, but will not be more than 6 tablespoons.

During the follow-up period we will watch your T-cells and your viral load closely. If at any time the study doctors think that you need treatment with antiretroviral medication, therapy will be started.

Everyone in the study should have a primary care physician. Your primary care physician will take care of any medical problems that you have that are not related to this study. With your permission, the study doctors will keep your primary care physician up-to-date of your progress in the study. If you do not have a primary care physician, we can refer you to one.

The results of all lab tests performed during the study will be shared with you, and (with your permission) with your primary care provider.

**Risks/Discomforts:**

The risks of this study include the side effects of the medicines used to treat HIV infection. There are currently more than 17 drugs licensed to treat HIV infection. The combinations of drugs used in this study will be selected according to the recommendations of the U.S. Department of Health and Human Services guidelines for the treatment of HIV infection.

The side effects of these drugs are well known and can include:

- nausea
- vomiting
- diarrhea
- headache
- fever
- chills
- fatigue
- malaise (flu-like symptoms)
- changes in liver, pancreas or kidney function
- decrease in the number or red blood cells (anemia)
- decrease in the number of white blood cells
- increase in the amount of “fat” in the blood (triglycerides)
- increased cholesterol
- increased blood sugar (diabetes)
- numbness and tingling in hands, feet or mouth
- difficulty concentrating
- grogginess or difficulty sleeping
- some people have a change in their body fat (lipodystrophy)
- some people may have allergic reactions to some medicines.

We will talk in detail with you the possible side effects of the medicines that you are going to take. You will be given written information about the drugs. It is possible that there are side effects to the medicines that are unknown at this time. We will inform you of any new information that we learn during the study.

There is a risk of increased side effects when taking a combination of drugs. There may also be other still unknown risks from taking these drugs in combination.

There is the risk of serious and/or life-threatening side effects when non-study medications are taken with study drugs. For your safety, you must tell your doctor or nurse about all the medications you are taking before you start the study and before taking any non-study medications while you are on the study. This includes over-the-counter medication, vitamins, herbal medications and nutritional supplements.

In addition, you must tell the study doctor or nurse before enrolling in any other clinical trials while you are on the study.

Another risk of taking HAART is the risk of developing resistance to medicines used to treat HIV infection. If you do not take your medicines properly, it is possible that the virus in your blood will mutate (change) and the medicines will not work well. This is called “resistance”. We know that the best way to avoid resistance is to take every dose of all of your medicines the way that we tell you. Therefore, we will make every effort to help you understand how to take your drugs. It is very important for you to let us know if you are having trouble with any of your medicines. The study staff will do everything possible to help you manage your side effects, and may change your medicines, if needed.

Drawing blood may cause mild pain, bleeding or bruising of your skin and, very rarely, infection. Some people become lightheaded or may faint when they have blood drawn.

**Risks of Pregnancy:**

Certain drugs used to treat HIV infection can harm a developing baby. You cannot join the study if you are pregnant. For that reason, if you are a woman who can have a baby, you will have a pregnancy test before you enter the study. We will give you another pregnancy test before you start HAART. You and your partner must use one method of birth control that you discuss with the study staff. You may choose one of the birth control methods listed below:

- male or female condoms with or without a cream or gel that kills sperm
- Diaphragm or cervical cap with a cream or gel that kills sperm
- Intrauterine device (IUD)

Some anti-HIV drugs require the use of two methods of birth control. If you are taking one of these medicines, the study staff will discuss your options with you. If you become pregnant while in the study we will review the medicines that you are taking. We may change the treatment to one that is approved for use in pregnant women.

**Benefits:**

The knowledge that we will gain from this study is very important. Your participation will help us to understand how to treat people with acute or early HIV infection. If you are randomized to receive treatment for your HIV infection, it is possible that you may benefit from this treatment. It is also possible that you will not benefit. Any treatment given to you will be tailored to your virus. This will be based on the results of the sensitivity test (genotype) that is done at your first study visit.

You may benefit from having very close follow-up of your HIV infection and frequent monitoring of your blood work. This follow-up and all study related lab tests will be provided at no cost to you.

**Costs to You:**

(Baltimore site)

We have asked the companies that make anti-HIV medications to donate drugs to our study participants. Some drugs may be available free of charge. However, it is possible that the results of your sensitivity test may show that you would respond better to a medicine that we do not have access to. In that case, we will ask that you use your health insurance to purchase that medicine. The study will reimburse you for any “co-pays” that you may have so that you will have no “out-of-pocket” expense. If you do not have health insurance, we will put you in contact with a social worker that can find out what services you qualify for.

(Canadian Sites)

Your medications will be provided to you at no charge.

**Alternatives to Participation:**

You may choose not to take medicines to treat HIV at this time. You may choose to receive treatment outside of this study. This choice is entirely up to you. Your decision will not affect your care at ___________________. You should not take part in this study if you know you definitely want to begin antiretroviral medicine at this time or if you know that you do not want to start taking antiretroviral medicine now. Remember, if you join this study, whether or not you receive antiretroviral medicines at this time will be determined by chance. The study staff will be happy to refer you to a doctor who can follow your HIV infection.

**Confidentiality:**

All information that you tell us will be kept confidential. All study data will be kept with a code or number. Your study records will be kept in a locked file cabinet in a locked office and/or on password protected computer files. No one will be able to see your study records except people working directly on the study. We will not give out any information about you unless you have given us written permission to do so.

The only people who will have access to this information will be those who are involved in the study. These people may include the researchers, study and lab personnel, and other study staff. Others who may see your information are the groups of people who make sure that the study is being done as it should be: the Committees on Human Research and staff, Audit and Compliance Officers, Legal Counsel and others including the Study Monitors who need to see your information to make sure that the study is going as planned.

Other groups of people who may be involved in the study and may need to see your information are:

- The government agency, the Office for Human Research Protection, that makes sure that we are conducting the research as planned, and the Food and Drug Administration.
- Doctors and staff at other places that are participating in this study.
- The sponsor of this study and people who the sponsor may contract with for this study. The name of the sponsor is National Institute of Allergy and Infectious Diseases.
- The Data Safety Monitoring Board.

(Baltimore Site only)

Maryland State Law requires us to report certain diseases to the Baltimore City Health Department. Any information relating to HIV infection will be reported using a unique identifier to ensure that your identity is kept private. Information that identifies you will not be given out to people who are not working on the study, unless you give us permission. If the study staff learns of possible child abuse and/or neglect, we will be required by Maryland State Law to tell the proper authorities.

At the end of the study, whatever we learn from the research may be published in a medical journal or used for teaching. Your name or other details about your health will not be used, so no one will be able to identify you personally.

**Leaving the Study Early:**

You can agree to be in the study now and change your mind later. If you wish to stop, please tell us right away. Leaving this study early will not stop you from getting regular medical care at _______________. If you leave the study early, we may use your health information that we already have if it is needed for this study or any follow-up activities. If you leave the study early and give us permission, we will call you a month after your last visit to see how you are doing. If you were taking HAART at the time that you leave the study we will call you a second time (2 months after your last visit).

**You may be taken out of the study if:**

- Staying in the study would be harmful to you.
- You need treatment not allowed in this study.
- You fail to follow instructions.
- The study is cancelled.
- You show bad behavior towards study or clinic staff.
- You are under the influence of drugs or alcohol during a study visit.
- There may be other reasons that we don’t know at this time to take you out of the study.

**Compensation:**

You will be paid $15.00 ($ 8.00 Canadian Sites) for each study visit. You will be paid about $240 ($128 Canadian Sites) for the first year. You will be paid about $60 ($32 Canadian Sites) per year for each additional year. If you take part in the study for all 3 years you may receive up to $360 ($192 Canadian Sites).

**Voluntariness:**

Your participation in this research project is completely voluntary. You have the right to withdraw from the study at any time. Even if you do not want to join the study, or if you withdraw from the study, you will still have the same quality of medical care available to you at _________________. You should ask the Principal Investigator listed below any questions you may have about this research study. You may ask him/her questions in the future if you do not understand something that is being done. The investigators will share with you any new findings that may be learned while you are taking part in this study.

**Research Related Injury:**

The ___________________________________________ and the _____________ government do not have any program to provide compensation to you if you experience injury, or other bad effects, which are not the fault of the investigators. If you feel that you have suffered an injury the study staff and/or the people in the Institutional Review Board office will answer your questions and/or help you find medical care.

**Persons to Contact:**

If you want to talk to anyone about this research study because you think you have not been treated fairly or think you have been hurt by joining the study, or you have any other questions about the study, you should call the Principal Investigator, **_________________** at **_______________** or call the Institutional Review Board at ____________. Either the Principal Investigator or the people in the Institutional Review Board office will answer your questions and/or help you find medical care if you feel you have suffered an injury.

If you have read this document and you have been given the chance to ask any questions now or at a later time or if the document has been read and explained to you and you agree to be in this study, please sign or make your mark below.

Print Name of Subject:_____________________________________________________

________________________________________________ _______________

Signature or Mark of Subject or Legally Authorized Date

Representative

________________________________________________ _______________

Signature of Person Obtaining Consent Date

________________________________________________ _______________

Witness to Consent if Subject Unable to Read or Write Date

*(Must be different than the person obtaining consent)*

Some of your blood will be kept in storage and tested after the study ends. Your blood sample will be labeled with a unique identifier (code number) and stored with this code number. Your blood sample will be linked to the other data collected about you in the study. We will not have your name on the blood sample. Your name will be kept in a locked file apart from your blood sample. Future testing may include studies to find out whether certain genetic factors influence how effective early treatment for HIV infection is. Stored blood will not be used for any research that is not related to this study.

You can still be in this study, even if you do not want your blood stored and tested for future studies.

I agree to storage of my blood sample for future testing

I do not agree to storage of my blood sample for future testing. If I do not allow my blood to be stored after the end of the study, I can still be in this study.

*If you agree to the storage of your blood now, but change your mind later, you may withdraw your consent at anytime. If you withdraw your consent to store blood, your blood samples will be destroyed. To withdraw your consent for future testing of stored blood please contact* ______________________ at ______________________

________________________________________________ _______________

Signature or Mark of Subject or Legally Authorized Representative Date

________________________________________________ _______________

Signature of Person Obtaining Consent Date

________________________________________________ _______________

Witness to Consent if Subject Unable to Read or Write Date

*(Must be different than the person obtaining consent)*

Note: Signed copies of this consent form must be: a) retained by the Principal Investigator, b) given to the participant, c) put in the patient’s medical record

APPENDIX II

SCHEDULE OF EVALUATIONS

|  | **Screening** | **Entry** | **Weeks** |  | **Months^(7)^** | |  |  |  |  |  |  |  |  |  |  |  |
| --- | --- | --- | --- | --- | --- | --- | --- | --- | --- | --- | --- | --- | --- | --- | --- | --- | --- |
| **Test** |  | Day 0 | 1 | 2 | 1 | 2 | 3 | 4 | 5 | 6 | 7 | 8 | 9 | 10 | 11 | 12^(4)^ | 15^(3)^ |
| **Medical History** | X |  | X* | X* | X* | X* | X* | X* | X* | X^*^ | X* | X* | X* | X* | X* | X* | X* |
| **Medication History** | X |  | X | X | X | X | X | X | X | X | X | X | X | X | X | X | X |
| **Randomization** |  | X |  |  |  |  |  |  |  |  |  |  |  |  |  |  |  |
| **Risk Behavior** | X |  | X | X | X | X | X | X | X | X | X | X | X | X | X | X | X |
| **Physical Exam** |  | X | X* | X* | X* | X* | X* | X* | X* | X* | X* | X* | X* | X* | X* | X | X* |
| **Adherence Questionnaire** |  |  | X | X | X | X | X | X | X | X | X | X | X | X | X | X | X |
| **CBC**** | 3 | 3 | 3 | 3 | 3 | 3 | 3 | 3 | 3 | 3 | 3 | 3 | 3 | 3 | 3 | 3 | 3 |
| **Chemistry Panel**** | 10 | 10 | 10 | 10 | 10 | 10 | 10 | 10 | 10 | 10 | 10 | 10 | 10 | 10 | 10 | 10 | 10 |
| **Lipid panel**** | 5 |  |  |  |  |  |  |  |  |  |  |  |  |  |  | 5 |  |
| **CD4+ cell count**** | 5 |  |  |  |  |  | 5 |  |  | 5 |  |  | 5 |  |  | 5 | 5^(6)^ |
| **Viral Load**** | 5 | 5 |  |  | 5 | 5 | 5 | 5 | 5 | 5 | 5 | 5 | 5 | 5 | 5 | 5 | 5^(6)^ |
| **HIV EIA/WB (2)** | X | X^(1)^ | X^(1)^ | X^(1)^ | X^(1)^ | X^(1)^ |  |  |  |  |  |  |  |  |  |  |  |
| **Repository**** | 50 |  |  |  | 50 | 10 | 50 | 50 | 10 | 50 | 10 | 50 | 10 | 50 | 10 | 50 | 50 |
| **Hepatitis B S AB^**^** | 2 |  |  |  |  |  |  |  |  |  |  |  |  |  |  |  |  |
| **Hepatitis B S Ag^**^** | 3 |  |  |  |  |  |  |  |  |  |  |  |  |  |  |  |  |
| **Hepatitis C ab^**^** | 2 |  |  |  |  |  |  |  |  |  |  |  |  |  |  |  |  |
| **Genotype**** | 5^(2)^ |  |  |  |  |  |  |  |  |  |  |  |  |  |  |  | 5^(2)^ |
| **TOTALS** | 83 | 18 | 13 | 13 | 68 | 23 | 73 | 68 | 28 | 73 | 23 | 63 | 23 | 63 | 23 | 78 | 73 |

* Vital Signs and Symptoms; interim medical history, symptom driven PE when necessary

**The numbers in the individual boxes indicate the amount of blood (in mL) that will be collected for the indicated test or procedure.

(1) If initially negative or indeterminate, will be repeated until fully positive

(2) Will be done on all patients at baseline, on anyone who experiences virologic failure while on treatment (as defined in section 5.2), and at month 15 on pts who discontinued HAART at month 12.

(3) After this time this visit will be repeated quarterly

(4) Months 12, 24, 36 and Treatment Discontinuation.

(6) CD4+ cell count and HIVRNA at months 18, 24, 30, 36

(7) Patients in whom treatment is initiated or re-initiated for clinical or immunologic reasons will be seen 2 weeks after treatment initiation, and monthly until their HIVRNA is <50 copies/mL at which time they will resume the visit schedule based on their date of entry.

APPENDIX III

DIVISION OF AIDS

## TABLE for GRADING SEVERITY of

ADULT ADVERSE EXPERIENCES ©

August, 1992

ABBREVIATIONS: Abbreviations utilized in the Table:

ULN = Upper Limit of Normal LLN = Lower Limit of Normal

Rx = Therapy Req = Required

Mod = Moderate IV = Intravenous

ADL = Activities of Daily Living Dec = Decreased

### ESTIMATING SEVERITY GRADE

For abnormalities NOT found elsewhere on the Toxicity Table, use the scale below to estimate grade of severity:

**GRADE 1 Mild** Transient or mild discomfort; no limitation in activity; no medical intervention/therapy required

**GRADE 2 Moderate** Mild to moderate limitation in activity - some assistance may be needed; no or minimal medical intervention/therapy required

**GRADE 3 Severe** Marked limitation in activity, some assistance usually required; medical intervention/therapy required, hospitalizations possible

**GRADE 4 Life-** Extreme limitation in activity, significant **threatening** assistance required; significant medical intervention/therapy required, hospitalization or hospice care probable

SERIOUS OR LIFE-THREATENING AEs

ANY clinical event deemed by the clinician to be serious or life-threatening should be considered a grade 4 adverse experience. Clinical events considered to be serious or life-threatening include, but are not limited to:seizures, coma, tetany, diabetic ketoacidosis, disseminated intravascular coagulation, diffuse petechiae, paralysis, acute psychosis

### MISCELLANEOUS When two values are used to define the criteria for each parameter, the lowest values will appear first.

> Parameters are generally grouped by body system.

> Some protocols may have additional protocol specific grading criteria.

Page 1 of 6

**PARAMETER GRADE 1 GRADE 2 GRADE 3 GRADE 4**

MILD MODERATE SEVERE POTENTIALLY LIFETHREATENING

**HEMATOLOGY**

Hemoglobin 8.0 g/dL - 9.4 g/dL 7.0 g/dL - 7.9 g/dL 6.5 g/dL- 6.9 g/dL <6.5 g/ dL

Absolute Neutrophil 1000 - 1500/mm^3^ 750 - 999/ mm^3^ 500 - 749/mm^3^ <500/ mm^3^

Count

Platelets 75,000 - 99,000/mm^3^ 50,000 - 74,999/mm^3^ 20,000 - 49,999/mm^3^ <20,000/mm^3^

Prothrombin Time(PT) >1.0 - 1.25 X ULN >1.25 - 1.5 X ULN >1.5 - 3.0 X ULN >3 X ULN

PTT >1.0 - 1.66 x ULN >1.66 - 2.33 x ULN >2.33 - 3.0 x ULN >3.0 x ULN

Methemoglobin 5.0 - 10.0% 10.1 - 15.0% 15.1 - 20.0% >20%

**CHEMISTRIES**

SODIUM

Hyponatremia 130 - 135 meq/L 123 - 129 meq/L 116 - 122 meq/L <116 meq/L

Hypernatremia 146 - 150 meq/L 151 - 157 meq/L 158 - 165 meq/L >165 meq/L

POTASSIUM

Hypokalemia 3.0 - 3.4 meq/L 2.5 - 2.9 meq/L 2.0 - 2.4 meq/L <2.0 meq/L

Hyperkalemia 5.6 - 6.0 meq/L 6.1 - 6.5 meq/L 6.6 - 7.0 meq/L >7.0 meq/L

PHOSPHATE

Hypophosphatemia 2.0 - 2.4 mg/dL 1.5 - 1.9 mg/dL 1.0 - 1.4 mg/dL <1.0 mg/dL

CALCIUM - (corrected for albumin)

Hypocalcemia 7.8 - 8.4 mg/dL 7.0 - 7.7 mg/dL 6.1 - 6.9 mg/dL <6.1 mg/dL

Hypercalcemia 10.6 - 11.5 mg/dL 11.6 - 12.5 mg/dL 12.6 - 13.5 mg/dL >13.5 mg/ dL

MAGNESIUM

Hypomagnesemia 1.2 - 1.4 meq/L 0.9 - 1.1 meq/L 0.6 - 0.8 meq/L <0.6 meq/ L

BILIRUBIN

Hyperbilirubinemia >1.0 - 1.5 x ULN >1.5 - 2.5 x ULN >2.5 - 5 x ULN >5 x ULN

GLUCOSE

Hypoglycemia 55 - 64 mg/dL 40 - 54 mg/dL 30 - 39 mg/dL <30 mg/ Dl

Page 2 of 6

**PARAMETER GRADE 1 GRADE 2 GRADE 3 GRADE 4**

MILD MODERATE SEVERE POTENTIALLY

LIFETHREATENING

Hyperglycemia 116 - 160 mg/dL 161 - 250 mg/dL 251 - 500 mg/dL >500 mg/ dL (nonfasting and

no prior diabetes)

Triglycerides ________ 400 - 750 mg/dL 751 - 1200 mg/dL >1200 mg/dL

Creatinine >1.0 - 1.5 x ULN >1.5 - 3.0 x ULN >3.0 - 6.0 x ULN >6.0 x ULN

URIC ACID

Hyperuricemia 7.5 - 10.0 mg/dL 10.1 - 12.0 mg/dL 12.1 - 15.0 mg/dL >15.0 mg/Dl

**LIVER TRANSAMINASE** (LFTs)

AST (SGOT) 1.25 - 2.5 x ULN >2.5 - 5.0 x ULN >5.0 - 10.0 x ULN >10.0 x ULN

ALT (SGPT) 1.25 - 2.5 x ULN >2.5 - 5.0 x ULN >5.0 - 10.0 x ULN >10.0 x ULN

GGT 1.25 - 2.5 x ULN >2.5 - 5.0 x ULN >5.0 - 10.0 x ULN >10.0 x ULN

Alk Phos 1.25 - 2.5 x ULN >2.5 - 5.0 x ULN >5.0 - 10.0 x ULN >10.0 x ULN

**PANCREATIC ENZYMES**

Amylase >1.0 - 1.5 x ULN >1.5 - 2.0 x ULN >2.0 - 5.0 x ULN >5.0 x ULN

Pancreatic amylase >1.0 - 1.5 x ULN >1.5 - 2.0 x ULN >2.0 - 5.0 x ULN >5.0 x ULN

Lipase >1.0 - 1.5 x ULN >1.5 - 2.0 x ULN >2.0 - 5.0 x ULN >5.0 x ULN

Page 3 of 6

**PARAMETER GRADE 1 GRADE 2 GRADE 3 GRADE 4**

MILD MODERATE SEVERE POTENTIALLY LIFETHREATENING

**CARDIOVASCULAR**

Cardiac Arrhythmia _____ Asymptomatic; Recurrent/ persistent Unstable dysrhythmia, transient dysrhythmia, dysrhythmia;symptomatic hospitalization,

no Rx req Rx req Rx req

Hypotension Transient orthostatic Symptoms correctable IV fluid req, Hospitalization req hypotension, no Rx with oral fluid Rx no hospitalization req

Hypertension Transient, increase Recurrent; chronic Acute Rx req; outpatient Hospitalization req >20 mm/ Hg; no Rx increase >20 mm/ Hg, hospitalization possible

Rx req

Pericarditis Minimal effusion Mild/ mod asymptomatic Symptomatic effusion, Tamponade- effusion, no Rx pain, EKG changes Pericardiocentesis OR surgery req

Hemorrhage, _____ Mildly symptomatic, Gross blood loss OR Massive blood loss no Rx required 1- 2 units transfused OR >2 units transfused

**GASTROINTESTINAL**

Nausea Mild OR transient; Mod discomfort OR Severe discomfort OR Hosptalization req reasonable intake intake decreased minimal intake for maintained for <3 days > 3 days

Vomiting Mild OR transient; Mod OR persistent; Severe vomiting of all Hypotensive shock OR 2- 3 episodes per day 4- 5 episodes per day; food/ fluids in 24 hrs hospitalization req OR mild vomiting OR vomiting lasting OR orthostatic hypotension IV Rx req

lasting <1 week > 1 week OR IV Rx req

Diarrhea Mild OR transient; Mod OR persistent; Bloody diarrhea; OR Hypotensive shock OR 3- 4 loose stools per 5- 7 loose stools per orthostatic hypotension hospitalization req day OR mild diarrhea day OR diarrhea OR >7 loose stools/ day

lasting <1 week lasting > 1 week OR IV Rx required

Oral Discomfort/ Mild discomfort, Difficulty swallowing Unable to swallow Unable to drink Dysphagia no difficulty but able to eat and solids fluids;IV fluids req swallowing drink

Constipation Mild Moderate Severe Distention with vomiting

Page 4 of 6

**PARAMETER GRADE 1 GRADE 2 GRADE 3 GRADE 4**

MILD MODERATE SEVERE POTENTIALLY LIFETHREATENING

**RESPIRATORY**

Cough (for Transient; no Rx Treatment associated Uncontrolled cough; _____

aerosol studies) cough; inhaled systemic Rx req

bronchodilator

Bronchospasm Acute Transient; no Rx; Rx req; normalizes No normalization Cyanosis; FEV1<25% FEV1 70% - <80% with bronchodilator; with bronchodilator; (or peak flow) OR (or peak flow) FEV1 50%-< 70% (or FEV1 25% - <50%(or intubated

peak flow) peak flow), retractions

Dyspnea Dyspnea on Dyspnea with normal Dyspnea at rest Dyspnea requiring

exertion activity O2 therapy

**NEUROLOGIC**

Neuro-cerebellar Slight incoordination Intention tremor OR Ataxia requiring Unable to stand

OR dysdiadochokinesia dysmetria OR slurred assistance to walk or

speech OR nystagmus arm incoordination

interfering with ADLs

Neuro- psych/ mood _______ _________ Severe mood changes Acute psychosis requiring medical req hospitalization intervention

Paresthesia Mild discomfort; no Mod discomfort; Severe discomfort;OR Incapacitating; (burning, Rx req non- narcotic narcotic analgesia req not responsive tingling, etc) analgesia req with symptomatic to narcotic improvement analgesia

Neuro- motor Mild weakness in Mod weakness in feet Marked distal weakness Confined to bed muscle of feet but (unable to walk on (unable to dorsiflex or wheel chair able to walk and/or heels and/ or toes), toes or foot drop) and because of

mild increase or mild weakness in mod proximal weakness muscle weakness

decrease in reflexes hands, still able to e.g., in hands interfering

do most hand tasks with ADLs and/ or requiring

and/or loss of assistance to walk and/ or

previously present unable to rise from chair

reflex or development unassisted

of hyperreflexia and/or

unable to do deep knee

bends due to weakness

Page 5 of 6

**PARAMETER GRADE 1 GRADE 2 GRADE 3 GRADE 4**

MILD MODERATE SEVERE POTENTIALLY LIFETHREATENING

Neuro- sensory Mild impairment (dec Mod impairment (mod Severe impairment Sensory loss involves sensation, e. g., dec sensation, e.g., (dec or loss of limbs and trunk. vibratory, pinprick, vibratory, pinprick, sensation to knees or

hot/cold in great toes) hot/cold to ankles) wrists) or loss of

in focal area or and/or joint position sensation of at least

symmetrical distri- or mild impairment mod degree in multiple

bution that is not different body areas

symmetrical (i. e., upper and lower

URINALYSIS extremities)

Proteinuria

Spot urine 1+ 2 - 3+ 4+ Nephrotic syndrome

24 hour urine 200 mg-1 g loss/ day OR >1 - 2 g loss/day OR >2 - 3.5 g loss/day OR Nephrotic syndrome <0.3% OR 0.3 - 1.0% OR >1.0% OR OR> 3.5 g loss/ day <3 g/l 3 - 10 g/l >10 g/ l

Gross Hematuria Microscopic only Gross, no clots Gross plus clots Obstructive OR transfusion req

**MISCELLANEOUS**

Fever 37.7 - 38.5C OR 38.6 - 39.5C OR 39.6 - 40.5C OR >40.5C OR

oral >12 hours 100.0 - 101.5F 101.6 - 102.9F 103 - 105F >105F

Headache Mild; no Rx req Mod; or non-narcotic Severe; OR responds to Intractable; or analgesia Rx initial narcotic Rx req repeated narcotic Rx

Allergic Reaction Pruritus without Localized urticaria Generalized urticaria Anaphylaxis rash angioedema

Cutaneous/Rash/ Erythema, pruritus Diffuse maculopapular Vesiculation OR ANY ONE:mucous Dermatitis rash OR dry desquamation moist desquamation membrane involvement, Or ulceration suspected Stevens- Johnson (TEN) erythema multiforme, necrosis req surgery, exfoliative dmatitis

Local Reaction Erythema Induration <10mm OR Induration >10mm OR Necrosis of skin

(2^o^ parenteral Rx - not inflammation OR ulceration

vaccination or skin test) phlebitis

Fatigue Normal activity Normal activity Normal activity reduced Unable to care reduced <25% reduced 25- 50% >50%; cannot work for self

Page 6 of 6

APPENDIX IV


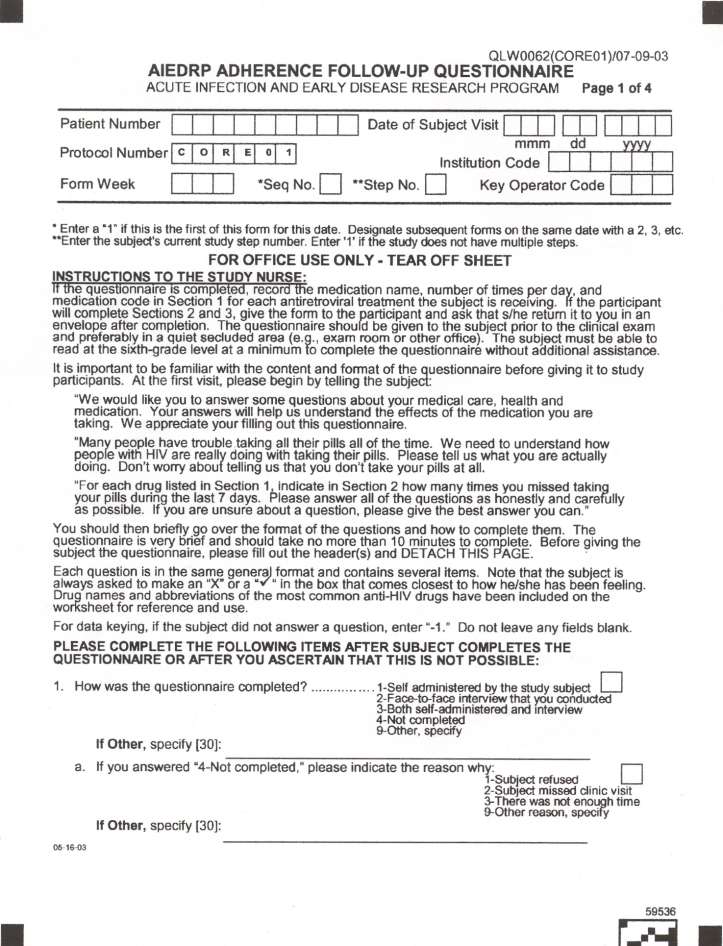

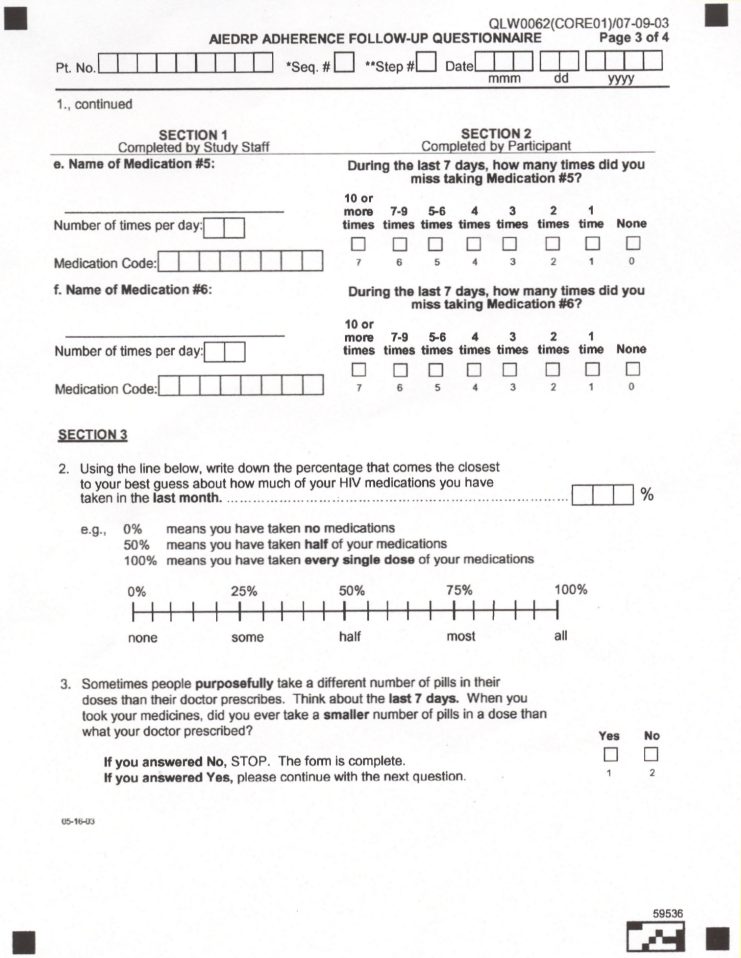

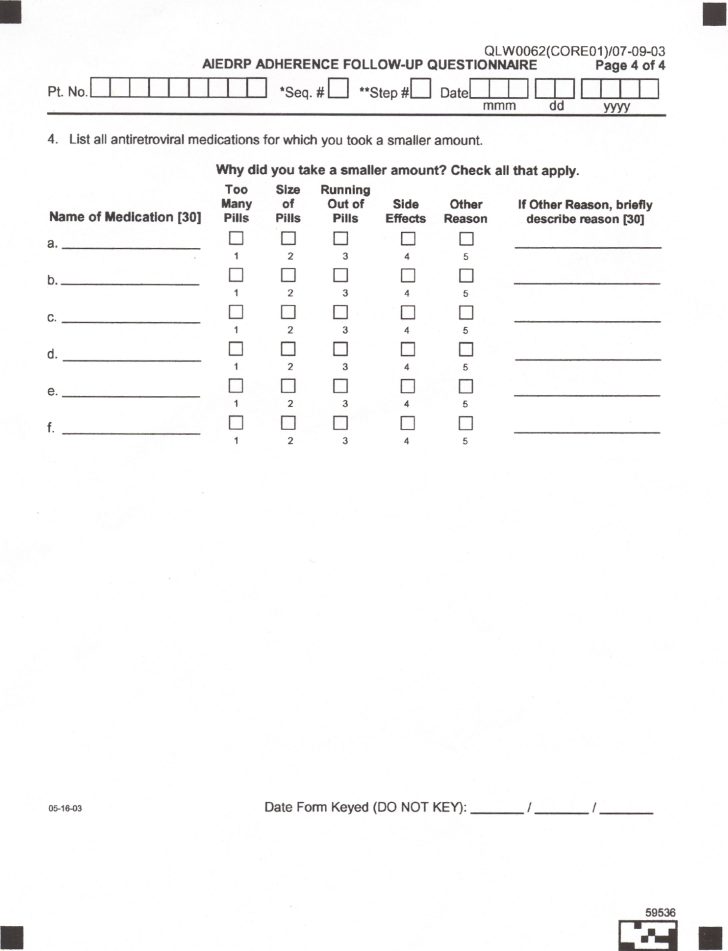


APPENDIX V

Staging HIV Infection

Each subject enrolled in the protocol will be classified based on their Seroconversion status at time of enrollment. The stages of HIV infection are as follows:

- **Pre-seroconversion antibody negative (Acute)**

Summary: Subjects with HIV infection demonstrated by HIV nucleic acid testing

or detection of HIV antigens prior to a reactive (positive) or indeterminate HIV

antibody test.

- **Pre-seroconversion Indeterminate antibody test (Acute)**

Summary: Subjects with HIV infection demonstrated by HIV nucleic acid testing

or detection of HIV antigens prior to a reactive (positive) but with an indeterminate HIV antibody test.

- **Peri-Seroconversion (Acute)**

Summary: Subjects with a reactive HIV antibody test, ≤ 60

days of antibody seroconversion.

- **Within 6 months of seroconversion (Early)**

Summary: Subjects with a reactive HIV antibody test, greater than

60 days to 6 months of antibody seroconversion.

- **6-12 months post-seroconversion (Early)**

Summary: Subjects between 6 and 12 months post-seroconversion
