# Supplementary material for: A Randomized Trial of Time-Limited Antiretroviral Therapy in Acute/Early HIV Infection
Source: PLoS One. 2015 Nov 24;10(11):e0143259. doi: 10.1371/journal.pone.0143259 (PMC4658016; doi:10.1371/journal.pone.0143259)
Supplement: S1 Table — (DOCX) [file pone.0143259.s005.docx]

## S1 Table. Association between successful completion of TL-cART and demographic variables

| **Variable** | **Completed and stopped ART (n=37)** | **Didn't stop or complete ART (n=20)** | **P^1^** | **Didn't complete ART (n=11)** | **Didn’t stop ART (n=9)** | **P^2^** | **P^3^** | **P^4^** |
| --- | --- | --- | --- | --- | --- | --- | --- | --- |
| Sex |  |  | 1.000 |  |  | 1.000 | 1.000 | 1.000 |
| Male | 34 (91.9) | 18 (90.0) |  | 10 (90.9) | 8 (88.9) |  |  |  |
| Female | 3 (8.1) | 2 (10.0) |  | 1 (9.1) | 1 (11.1) |  |  |  |
| Stratum |  |  | 0.728 |  |  | 1.000 | 1.000 | 0.645 |
| Acute | 6 (16.2) | 4 (20.0) |  | 2 (18.2) | 2 (22.2) |  |  |  |
| Early | 31 (83.8) | 16 (80.0) |  | 9 (81.8) | 7 (77.8) |  |  |  |
| Race |  |  | 0.157 |  |  | 0.244 | 0.028 | 0.798 |
| Black or African American | 9 (24.3) | 9 (45.0) |  | 6 (54.5) | 3 (33.3) |  |  |  |
| White | 26 (70.3) | 9 (45.0) |  | 3 (27.3) | 6 (66.7) |  |  |  |
| Other | 2 (5.4) | 2 (10.0) |  | 2 (18.2) | 0 (0.0) |  |  |  |
| Ethnicity |  |  | 0.119 |  |  | 1.000 | 0.410 | 0.357 |
| Hispanic or Latino | 1 (2.7) | 0 (0.0) |  | 0 (0.0) | 0 (0.0) |  |  |  |
| Not Hispanic or Latino | 36 (97.3) | 18 (90.0) |  | 10 (90.9) | 8 (88.9) |  |  |  |
| Not available to clinic | 0 (0.0) | 2 (10.0) |  | 1 (9.1) | 1 (11.1) |  |  |  |
| Route of transmission |  |  |  |  |  |  |  |  |
| Injection drug use | 9 (25.0) | 17 (89.5) | 0.295 | 2 (20.0) | 0 (0.0) | 0.474 | 1.000 | 0.169 |
| MSM | 30 (81.1) | 14 (70.0) | 0.509 | 7 (63.6) | 7 (77.8) | 0.642 | 0.246 | 1.000 |
| Heterosexual contact | 8 (21.6) | 5 (25.0) | 0.754 | 3 (27.3) | 2 (22.2) | 1.000 | 0.697 | 1.000 |
| Age (yr) |  |  | 0.016 |  |  | 0.403 | 0.020 | 0.170 |
| Median (IQR) | 36.8 (31.7, 46.5) | 27.7 (24.5, 35.2) |  | 27.0 (20.6, 35.7) | 29.5 (26.1, 34.7) |  |  |  |
| Mean (SD) | 38.0 (11.3) | 30.7 (9.7) |  | 28.8 (8.0) | 33.0 (11.6) |  |  |  |
| Range | (19.3, 58.4) | (19.0, 56.1) |  | (19.0, 41.9) | (21.6, 56.1) |  |  |  |
| Baseline CD4 cell count (/ul) |  |  | 0.707 |  |  | 0.970 | 0.615 | 0.956 |
| Median (IQR) | 500 (382, 650) | 533 (430, 653) |  | 506 (440, 650) | 572 (400, 662) |  |  |  |
| Mean (SD) | 528.2 (180.5) | 535.5 (135.3) |  | 547.9 (116.8) | 520.2 (161.1) |  |  |  |
| Range | (237, 900) | (220.0, 770.0) |  | (420, 770) | (220, 676) |  |  |  |
| Baseline CD8 cell count (/ul) |  |  | 0.345 |  |  | 0.790 | 0.492 | 0.430 |
| Median (IQR) | 940 (772, 1240) | 880 (705, 1045) |  | 850 (678, 1320) | 910 (745, 950) |  |  |  |
| Mean (SD) | 1180.3 (1019.7) | 905.2 (286.9) |  | 941.2 (325.1) | 861.1 (243.7) |  |  |  |
| Range | (166, 6369) | (530.0, 1508.0) |  | (556, 1508) | (530, 1290) |  |  |  |
| Baseline viral load (log_10_ copies/ml) |  |  | 0.880 |  |  | 0.970 | 0.883 | 0.934 |
| Median (IQR) | 4.6 (4.2, 5.0) | 4.7 (4.2, 4.9) |  | 4.7 (4.5, 4.9) | 4.7 (4.1, 5.1) |  |  |  |
| Mean (SD) | 4.7 (0.8) | 4.6 (0.6) |  | 4.6 (0.4) | 4.6 (0.9) |  |  |  |
| Range | (3.1, 6.6) | (3.5, 5.9) |  | (3.8, 5.0) | (3.5, 5.9) |  |  |  |

1. Comparison between “completed and stopped” and “didn’t stop/complete”
2. Comparison between “didn’t complete” and “didn’t stop”
3. Comparison between “completed and stopped” and “didn’t complete”

Comparison between “completed and stopped” and “didn’t stop”
